# A distinct metabolic state arises during the emergence of 2‐cell‐like cells

**DOI:** 10.15252/embr.201948354

**Published:** 2019-12-18

**Authors:** Diego Rodriguez‐Terrones, Götz Hartleben, Xavier Gaume, André Eid, Manuel Guthmann, Ane Iturbide, Maria‐Elena Torres‐Padilla

**Affiliations:** ^1^ Institute of Epigenetics and Stem Cells Helmholtz Zentrum München München Germany; ^2^ Institute for Diabetes and Cancer German Center for Diabetes Research Helmholtz Zentrum München Neuherberg Germany; ^3^ Faculty of Biology Ludwig‐Maximilians Universität München Germany

**Keywords:** 2‐cell‐like cells, metabolism, pluripotency, reprogramming, totipotency, Metabolism, Regenerative Medicine

## Abstract

Pluripotent stem cells are thought of as a surrogate of early developmental stages that sustain the capacity to generate all cell types in the body, thereby constituting an invaluable tool to address the mechanisms underlying cellular plasticity. In the mouse, cells resembling totipotent 2‐cell‐stage embryos (2‐cell‐like cells) arise at a very low frequency in embryonic stem cell (ESC) cultures. However, the extent to which these early‐embryonic‐like cells recapitulate the molecular features of the early embryo is unclear. Here, we have undertaken a characterization of some of the metabolic features of early‐embryonic‐like cells in culture. Our data indicate that early‐embryonic‐like cells exhibit decreased glycolytic and respiratory activity, lower levels of reactive oxygen species and increased glucose uptake, suggesting a shift of the metabolic programme during 2‐cell‐like cell reprogramming. Accordingly, we find that 2‐cell‐like cells can be induced by defined metabolites. Thus, in addition to their transcriptional and chromatin features, 2‐cell‐like cells recapitulate some of the metabolic features of their *in vivo* counterpart. Altogether, our work underscores a distinct metabolic state of early‐embryonic‐like cells and identifies compounds that can induce their emergence *in vitro*.

## Graphical abstract

2‐cell‐like cells share some metabolic features with the early mouse embryo, indicating a shift of the metabolic programme during 2‐cell‐like cell reprogramming.

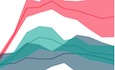

## Introduction

The metabolic state of a cell is a key feature of cellular identity and has been linked to cellular plasticity. Shifts in metabolic pathways and reactive oxygen species (ROS) have been involved in reprogramming cell fate 1. Likewise, the Warburg effect, whereby aerobic glycolysis becomes predominant over oxidative phosphorylation, is a well‐known feature of cancer cells which is thought to satisfy the altered metabolic demands that arise upon cellular transformation 2. The importance of cellular metabolism during changes in cell fate is therefore beginning to emerge, particularly because it opens up the possibility to manipulate cell fate through inducing changes in metabolic programmes.

Pluripotent embryonic stem cells (ESCs) derived from the inner cell mass of the mouse blastocyst can self‐renew indefinitely, provided appropriate culture conditions 3. Mouse ESCs are pluripotent, since they have the capacity to generate all the cells in the body, including the germline, when transplanted into blastocysts to form chimera. ESC cultures are heterogeneous and are known to harbour different cellular states which vary depending on the culture conditions used 4, 5, 6. Namely, when grown in serum and LIF, ESCs fluctuate between a naïve state, which is considered reminiscent of the pre‐implantation epiblast, and a primed state, closer in nature to the post‐implantation epiblast. The latter has a limited capacity to contribute to chimeras and the germline, compared to naïve ESCs 7. These two cell populations recapitulate several molecular features of their *in vivo* counterparts, including their DNA methylation profiles 8, the expression of pluripotency markers 9 and their metabolic state 10. Whereas naïve pluripotent stem cells rely on a mixture of glycolytic and aerobic metabolism, primed pluripotent stem cells rely almost exclusively on glycolysis to satisfy their energetic demands. In other words, naïve mouse ESCs respire more than the more primed EpiSCs 10. Thus, there appears to be a link between the maintenance and loss of pluripotency, and the state of cellular metabolism.

In addition to the aforementioned heterogeneities of naïve and primed ESCs, cells resembling the blastomeres of the 2‐cell‐stage embryo have been documented to arise spontaneously in these cultures 11. These “2‐cell‐like cells” constitute ~ 0.5% of the mouse ESC culture and display transcriptional and chromatin accessibility profiles highly similar to those in the 2‐cell‐stage embryo 11, 12, 13, as well as greater histone mobility 14 and dispersed chromocentres 15, all of which are molecular features characteristic of the 2‐cell‐stage embryo. In addition, 2‐cell‐like cells display expanded cellular potency and higher reprogrammability upon somatic cell nuclear transfer 11, 15, underscoring their broader plasticity. Two‐cell‐like cells emerge from cells that express the transcription factor Zscan4 (Zscan4^+^ cells) 16, which are yet another subpopulation of ESC cultures constituting approximately 5% of the cell population 17, 18. Early‐embryonic‐like cells (Zscan4^+^ and 2‐cell‐like cells) can be induced in culture through the modulation of specific chromatin pathways, including the chromatin assembly factor 1 (CAF‐1) 15 and the non‐canonical polycomb repressive complex PRC1.6 16, 19, as well as the transcription factors Dux and Dppa2/4 12, 20, 21, 22.

Pre‐implantation mouse embryos up to the 8‐cell stage rely exclusively on monocarboxylates such as pyruvate and lactate to satisfy their bioenergetic needs 23, 24, 25. This contrasts to morula and blastocyst‐stage embryos, which rely on glucose to produce energy through a combination of glycolysis and oxidative phosphorylation 23, 24. Thus, there is a switch in central carbon metabolism as development proceeds, when the embryo transits from a totipotent, to a more restricted, pluripotent stage. Stem cells maintained *in vitro* may recapitulate some of their counterparts *in vivo*. However, it is unclear whether the different cellular heterogeneities in ESCs also reflect changes in metabolic pathways. In particular, whether 2‐cell‐like cells recapitulate some of the metabolic characteristics of 2‐cell‐stage embryos has not been investigated.

Here, we set out to investigate whether 2‐cell‐like cells display different metabolic features, compared to ESCs. We show that 2‐cell‐like cells display lower glycolytic and respiratory activity. Notably, this metabolic shift occurs in concert with a marked change in mitochondrial morphology, a significant reduction in ROS levels and a considerable increase in glucose uptake, suggesting a remodelling of metabolic activity upon 2‐cell‐like cells emergence. Importantly, Zscan4^+^ cells display mostly intermediate metabolic features, between 2‐cell‐like cells and ESCs, suggesting gradual metabolic reprogramming during the transition from ESCs to 2‐cell‐like cells. Finally, by carrying out a small‐scale metabolite screen, we identified three compounds that promote the spontaneous emergence of early‐embryonic‐like cells in a dose‐dependent fashion. Overall, our data indicate that 2‐cell‐like cells transition into an overall “quiet” metabolic state and identify specific metabolites that induce them in culture.

## Results and Discussion

**Figure 1:**
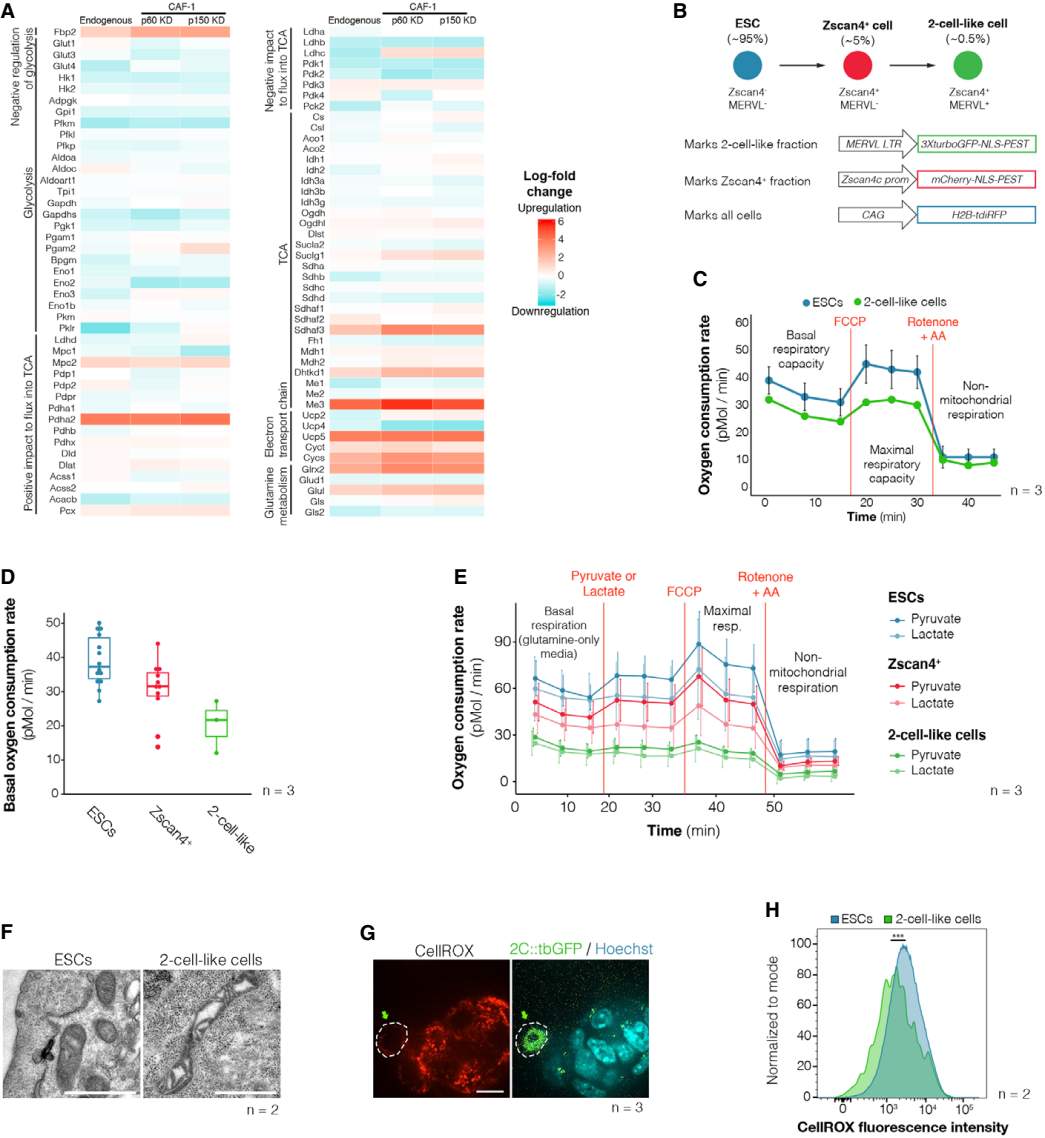
Early‐embryonic‐like cells exhibit decreased mitochondrial respiration Heatmap showing changes in RNA expression levels for various enzymes and regulators of central carbon metabolism in endogenous and in CAF1‐knockdown‐induced (through p60 or p150 KD) 2‐cell‐like cells. Fold‐changes relative to ESCs were calculated based on bulk RNA‐seq data 15.Schematic representation of 2‐cell‐like cell emergence from ES cells, which transit through the intermediate Zscan4^+^ state before becoming 2‐cell‐like cells. Reporter constructs used to identify the three cell populations are shown.Oxygen consumption rate of ES (blue line) and 2‐cell‐like cells (green line). Assay medium was formulated to recapitulate standard ES cell culture conditions and contained glucose, l‐glutamine and pyruvate. Basal, maximal (FCCP‐induced) and non‐mitochondrial (rotenone and antimycin A‐mediated) respiratory rates are indicated. A representative graph of three independent biological replicates performed on the Seahorse extracellular flux analyser is shown. Due to the low number of 2‐cell‐like cells available, compared to ESCs, one technical replicate of the former was analysed per biological replicate, while three or more technical replicates were performed for the latter. Accordingly, mean ± s.d. of technical replicates is shown for ESCs.Basal oxygen consumption rate of ES (blue), Zscan4^+^ (red) and 2‐cell‐like cells (green) across three independent biological replicates performed on the Seahorse extracellular flux analyser. Assay medium was formulated to recapitulate standard ES cell culture conditions and contained glucose, l‐glutamine and pyruvate. Boxes indicate the range between the first and third quartile, the band specifies the median, and the whiskers span the range of the data while extending no further than 1.5 times the interquartile range. Individual dots indicate the measurements obtained in each of the individual technical replicates.Oxygen consumption rate of ES (blue line), Zscan4^+^ (red line) and 2‐cell‐like cells (green line) in glucose‐free media and upon acute injection of sodium pyruvate or sodium l‐lactate. Note that l‐glutamine—but not glucose or pyruvate—was initially present in the assay medium. Maximal (FCCP‐induced) and non‐mitochondrial (rotenone and antimycin A‐mediated) respiratory rates following pyruvate or lactate treatment are also indicated. A graph including data from three independent biological replicates is presented, and the mean ± s.d. of technical replicates is shown.Representative electron micrographs of mitochondria from ES (*n* = 49 sections) and 2‐cell‐like cells (*n* = 57 sections) generated across two independent technical and biological replicates. Scale bar, 1 μm. Representative images from two independent biological and technical replicates are shown. 162 and 99 mitochondria were analysed for 2‐cell‐like and ES cells, respectively.Representative single section of CellROX‐DeepRed fluorescence in ES and 2‐cell‐like cells (green arrow) obtained using live‐cell microscopy. Scale bar, 10 μm. Representative images from three independent biological replicates are shown.FACS‐assisted quantification of CellROX‐DeepRed fluorescence intensity in ES and 2‐cell‐like cells. Measurements were obtained from two independent biological replicates. ***P < 0.005; Mann–Whitney *U* test. Source data are available online for this figure.

We first interrogated our previously reported RNA‐seq datasets 15 for changes in the expression levels of genes involved in metabolic regulation (Fig 1A). We compared expression levels between ESCs and 2‐cell‐like cells obtained through three distinct means, namely: spontaneously arising 2‐cell‐like cells (endogenous 2‐cell‐like cells) and CAF‐1 knockdown‐induced 2‐cell‐like cells obtained upon depletion of either of the two main subunits of CAF‐1 (p60 and p150) 15. We determined expression changes for most major central carbon metabolism enzymes and regulators, which we broadly classified into four groups: those involved in glycolysis, the TCA cycle, electron transport or glutamine metabolism (Fig 1A). Globally, while glycolytic enzymes displayed a tendency to be downregulated, we did not detect major changes in the expression of TCA cycle enzymes themselves (Fig 1A). In addition, several genes whose activity would be predicted to promote metabolic flux into the TCA cycle were upregulated in 2‐cell‐like cells, while others whose activity is known to strongly inhibit TCA cycle flux, including PDK and LDH, were downregulated (Fig 1A). Our analysis revealed that overall, 2‐cell‐like cells display marked differences in the expression levels of several enzymes and regulators involved in central carbon metabolism. These findings suggest potential changes in the metabolic activity of 2‐cell‐like cells compared to ESCs. Notwithstanding, because metabolic flux cannot be robustly predicted based on gene expression data alone—mainly due to the fact that metabolic changes are primarily regulated through modulation of enzymatic activity, for example through post‐translational modifications and changes in substrate concentration—we set out to investigate the respiratory capacity of 2‐cell‐like cells directly.

**Figure EV1:**
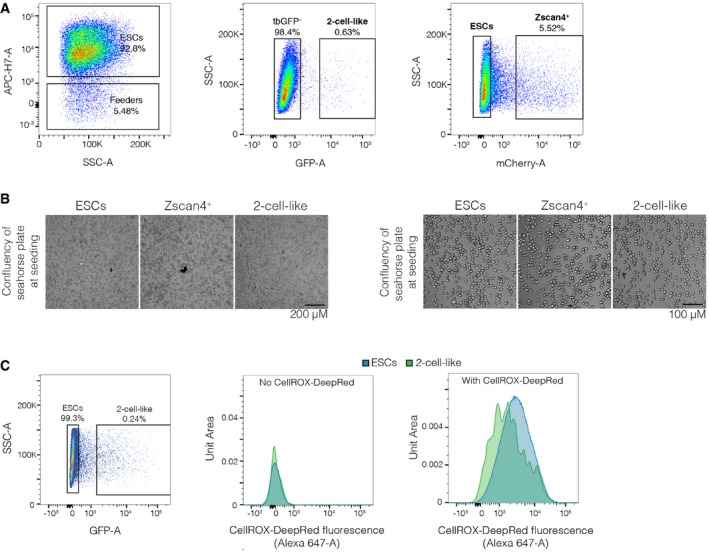
Controls and set‐up for Seahorse and ROS measurements Representative sorting gates used for the isolation of ES, Zscan4^+^ and 2‐cell‐like cells used throughout this study. Feeder cells were removed on the basis of their lack of far‐red fluorescence, which is higher in ES cells because of the presence of an H2B‐iRFP cassette. ESCs were defined as double negative for both the Zscan4 (Zscan4c::mCherry^−^) and the MERV‐L reporters (2C::tbGFP^−^). Zscan4^+^ cells were defined as positive for the Zscan4 reporter but negative for the MERV‐L reporter, and 2‐cell‐like cells were defined as positive for both reporters.Brightfield microscopy images indicating the confluency of the three populations shortly after plating in the Seahorse extracellular flux analyser plates. Representative images, in two magnifications, for the three independent biological replicates presented in Fig 1C and D are shown.Representative sorting gate for ES and 2‐cell‐like cells (left) used for the FACS‐assisted ROS measurements (right). Fluorescence intensity distributions for ES cells (blue) and 2‐cell‐like cells (green) in control (centre) and CellROX‐treated samples (right) are shown.

**Figure EV2:**
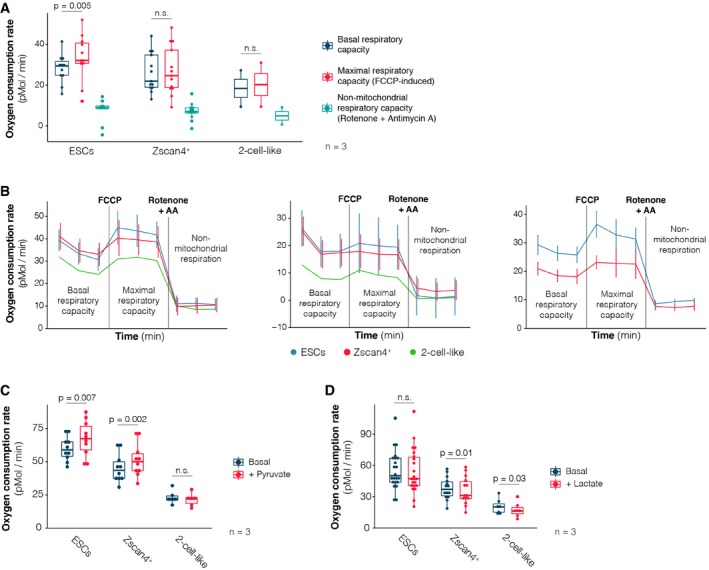
Seahorse oxygen consumption rate measurements ABasal, maximal (FCCP‐induced) and non‐mitochondrial (rotenone + antimycin A‐induced) oxygen consumption rate measurements of ES, Zscan4^+^ and 2‐cell‐like cells performed on the Seahorse extracellular flux analyser. Assay medium was formulated to recapitulate standard ES cell culture conditions and contained glucose, l‐glutamine and pyruvate. Measurements were carried out in three independent biological replicates. Note that 2‐cell‐like cells could only be profiled in two of those replicates. *P*‐value corresponds to a paired *t*‐test.BAdditional individual replicates of the oxygen consumption rate measurements of ESCs (blue line), Zscan4^+^ (red line) and 2‐cell‐like cells (green line) measured on the Seahorse extracellular flux analyser, as in Fig 1C. Basal, maximal (FCCP‐induced) and non‐mitochondrial (rotenone‐mediated) respiratory rates are indicated. Due to the low number of 2‐cell‐like cells in ESC cultures compared to ES and Zscan4^+^ cells, one technical replicate of the former was analysed per biological replicate, while three technical replicates were performed for the two other populations. Accordingly, mean ± s.d. of technical replicates is shown for ES and Zscan4^+^ cells.C, DOxygen consumption rate measurements of ES, Zscan4^+^ and 2‐cell‐like cells performed on the Seahorse extracellular flux analyser. Basal measurements in glucose‐free media and upon acute injection of sodium pyruvate (C, 20 mM) or sodium l‐lactate (D, 20 mM) are shown. Note that l‐glutamine—but not glucose or pyruvate—was initially present in the assay medium. Measurements from three independent biological replicates are shown. *P*‐values correspond to paired *t*‐tests.Data information: Boxes indicate the range between the first and third quartile, the band depicts the median, and the whiskers span the range of the data while extending no further than 1.5 times the interquartile range. Individual dots indicate the measurements obtained in each technical replicate. Source data are available online for this figure.

Because of the limiting amounts of 2‐cell‐like cells available through cell sorting, we were unable to perform metabolomic profiling of these cells. Instead, to directly assess whether early‐embryonic‐like cells exhibit an overall distinct pattern of metabolic activity than ESCs, we first measured oxygen consumption in 2‐cell‐like cells. We used the Seahorse extracellular flux analyser to measure the oxygen consumption rate (OCR) in live cells and determine basal, maximal and non‐mitochondrial respiration. We optimized conditions for low cell numbers, which we set at 50,000 cells per well. Using a previously described reporter cell line (Fig 1B and Tables 1 and 2), we FACS‐sorted equal numbers of ESCs (Zscan4c::mCherry^–^, 2C::tbGFP^–^) and 2‐cell‐like cells (Zscan4c::mCherry^+^, 2C::tbGFP^+^) and profiled them on the Seahorse analyser (Fig EV1A and B). We used medium containing 25 mM glucose, 1 mM pyruvate and 2 mM glutamine, which is equivalent to the standard concentrations in ESC culture medium. Two‐cell‐like cells displayed a lower basal oxygen consumption rate compared to ESCs (Fig 1C), indicating a decrease in mitochondrial respiration. When challenged with FCCP, which uncouples the proton gradient from oxidative phosphorylation in mitochondria to reveal maximal respiratory capacity, ESCs augmented their oxygen consumption rate (Figs 1C, and EV2A and B). However, this was not the case for 2‐cell‐like cells, which remained at similar OCR levels compared to basal conditions (Fig 1C, and EV2A and B). We observed no differences in OCR between these two cell types after rotenone addition, suggesting similar levels of extra‐mitochondrial oxygen consumption rates (Fig EV2A and B). Interestingly, Zscan4^+^ cells (Zscan4c::mCherry^+^, 2C::tbGFP^–^) displayed intermediate levels of basal and maximal respiratory capacity, compared to ESCs and 2‐cell‐like cells (Figs 1D, and EV2A and B), in agreement with their intermediate nature during the transition of ESCs to the 2‐cell‐like state 16. Of note, oligomycin treatment was not tolerated by 2‐cell‐like cells in this experimental set‐up, which prevented us from determining the levels of ATP‐linked respiration. Altogether, these results indicate that 2‐cell‐like cells display lower cellular respiratory capacity than ESCs and that in basal conditions, 2‐cell‐like cells respire at maximum capacity.

**Table 1:** Reporter cell lines used in this study

Cell line	Green channel	Red channel	Far‐red channel	Described in
tbg4	2C::3XturboGFP‐NLS‐PEST			Ishiuchi *et al* 15
tbg4‐12	2C::3XturboGFP‐NLS‐PEST	CAG::NLS‐tdTomato (constitutive)		Rodriguez‐Terrones *et al* 16
tbg4ZH	2C::3XturboGFP‐NLS‐PEST	Zscan4c::mCherry‐NLS‐PEST	CAG::H2B‐tdiRFP (constitutive)	Rodriguez‐Terrones *et al* 16
2C‐EGFP	2C::EGFP			Ishiuchi *et al* 15
Rex1‐Zscan4	Rex1::EGFP‐PEST (knock‐in)	Zscan4c::tdTomato‐PEST		Rodriguez‐Terrones *et al* 16
2C‐tdTomato		2C::tdTomato	CAG::H2B‐tdiRFP (constitutive)	Ishiuchi *et al* 15

The lower respiratory capacity of Zscan4^+^ and 2‐cell‐like cells compared to ESCs prompted us to investigate whether this decrease could be attributed to different substrate preferences in Zscan4^+^ and 2‐cell‐like cells. Because 2‐cell‐stage embryos rely on monocarboxylates such as pyruvate or lactate, we next measured the respiratory response of ES, Zscan4^+^ and 2‐cell‐like cells to acute supplementation of these two metabolites. For these experiments, we used medium without glucose and pyruvate, but containing l‐glutamine to sustain a basal level of respiration. ES and Zscan4^+^ cells increased their oxygen consumption upon pyruvate supplementation, but 2‐cell‐like cells did not (Figs 1E and EV2C). None of the three cell types increased their oxygen consumption rate upon lactate supplementation (Fig EV2D). These observations may reflect the inability of 2‐cell‐like cells to take up exogenous pyruvate and/or the fact that 2‐cell‐like cells are already respiring at maximal capacity.

**Figure EV3:**
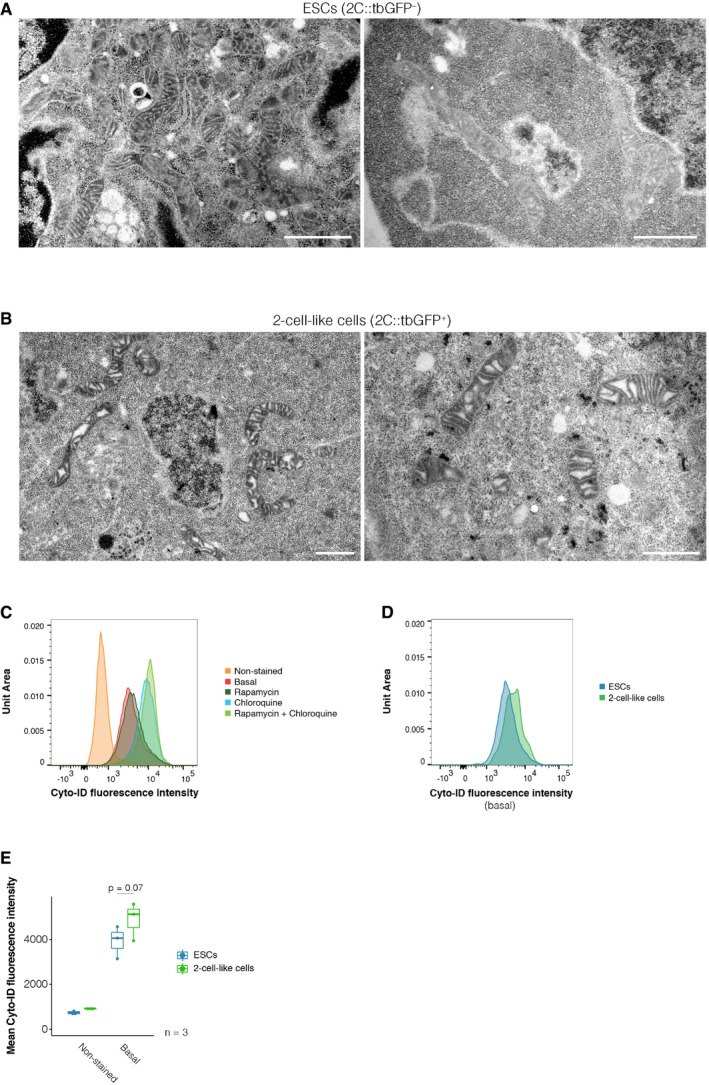
Electron micrographs of mitochondria in ES cells and 2‐cell‐like cells A, BRepresentative electron micrographs of mitochondria from ES and 2‐cell‐like cells. Scale bars, 1 μm.CAutophagic flux measurements were carried out using Cyto‐ID. Non‐stained cells were employed as a negative control and show a distinct fluorescent profile. Chloroquine‐ and/or rapamycin‐treated cells were used as a positive control and exhibited increased fluorescence intensity.DFACS‐assisted measurement of Cyto‐ID fluorescence intensity in ES (blue) and 2‐cell‐like cells (green).EQuantification of Cyto‐ID fluorescence intensity in ES (blue) and 2‐cell‐like (green) cells. Boxes indicate the range between the first and third quartile, the band indicates the median, and the whiskers span the range of the data while extending no further than 1.5 times the interquartile range. Individual dots indicate the median fluorescence intensity measurements obtained in each biological replicate. *P*‐values were calculated using a paired *t*‐test. Source data are available online for this figure.

**Figure EV4:**
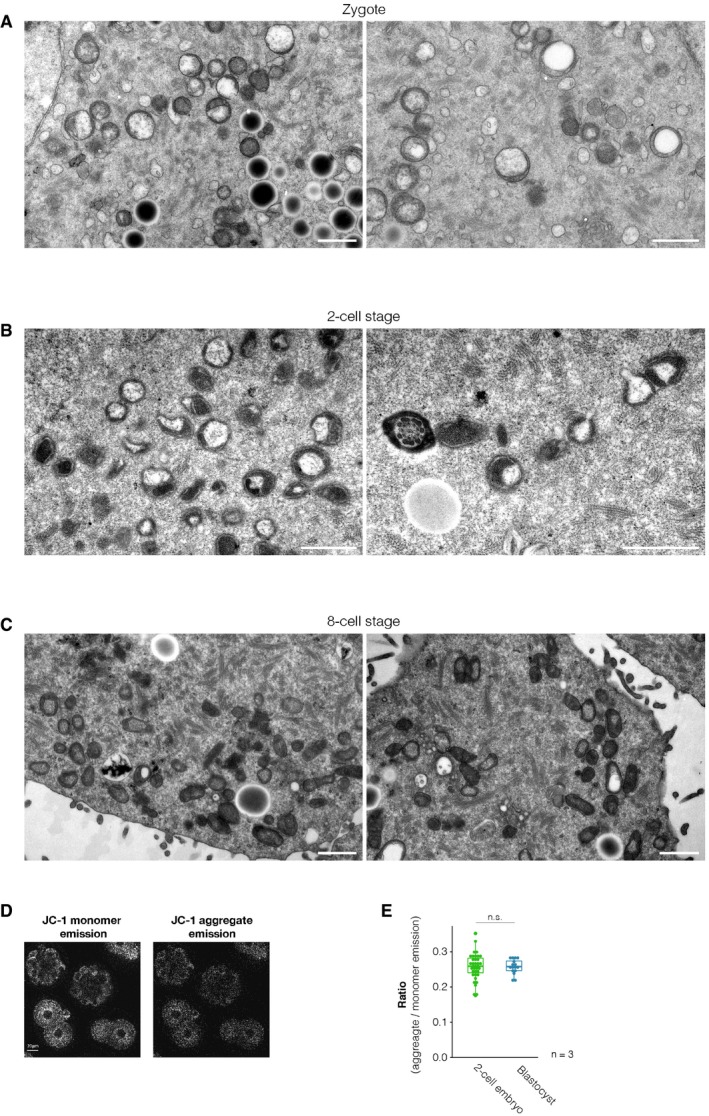
Electron micrographs of mitochondria from pre‐implantation embryos A–CRepresentative electron micrographs of mitochondria from PN3 stage zygotes and 2‐cell‐ and 8‐cell‐stage blastomeres. Scale bars, 1 μm.DLive‐cell imaging of mouse 2‐cell‐stage embryos and blastocysts stained with the mitochondrial membrane potential probe JC‐1.EQuantification of the ratio between aggregate and monomer emission in mouse 2‐cell‐stage embryos and blastocysts. Boxes indicate the range between the first and third quartile, the band specifies the median, and the whiskers span the range of the data while extending no further than 1.5 times the interquartile range. Measurements were obtained in three independent biological replicates, and each dot represents one individual embryo (*n* = 34, 2‐cell stage; *n* = 16, blastocyst). n.s.—non‐significant; *t*‐test.

The observation that maximal respiratory capacity decreases in 2‐cell‐like cells raised the possibility that mitochondrial architecture might change upon reprogramming to the 2‐cell‐like state. To test this hypothesis, we examined mitochondrial morphology in ESCs and 2‐cell‐like cells by electron microscopy (Figs 1F, and EV3A and B). Two‐cell‐like cells contained a larger proportion of elongated mitochondria in comparison with ESCs (25% of mitochondria were longer than 1.5 μm in 2‐cell‐like cells, versus only 9% in ES cells, *n* = 162 and 99 mitochondria, respectively). Instead of the more developed cristae typical of serum/LIF‐grown ESCs 10, 26, 2‐cell‐like cells exhibited mitochondria with a matrix that was electron poor and tended to exhibit irregularly folded cristae (Fig 1F), in agreement with their overall lower maximal respiratory capacity. Mitochondria with irregularly folded cristae have been associated with lower oxygen consumption 27, 28. The electron micrographs also suggested increased vacuolization in the cytoplasm of 2‐cell‐like cells, which prompted us to measure autophagy. We found that 2‐cell‐like cells display slightly higher levels of autophagic vesicles, as measured by Cyto‐ID fluorescence (Fig EV3C–E). However, this difference was not statistically significant. The analysis of additional autophagic markers is necessary to address the biological relevance of these changes. In agreement with previous reports 28, electron micrographs of pre‐implantation embryos showed that mitochondria in the zygote and 2‐cell‐stage embryo also possess an electron‐poor matrix with concentrically organized cristae around it (Fig EV4A and B), as opposed to the more opaque matrix observed already at the 8‐cell stage (Fig EV4C) or the transverse cristae observed in blastocysts 28 and ES cells (Figs 1F and EV3A). While the mitochondria of 2‐cell‐like cells also display increased electron‐poor matrix volume, they may not fully recapitulate the mitochondrial morphology of the 2‐cell embryo. Further studies will be needed to assess the biological relevance of these changes. Indeed, despite the known differences in mitochondrial morphology between 2‐cell embryos and blastocysts, we did not detect changes in mitochondrial membrane potential, as assayed using JC‐1 staining (Fig EV4D and E).

As an additional indicator of mitochondrial activity, we measured levels of reactive oxygen species (ROS) in 2‐cell‐like cells, since altered ROS levels are often indicative of altered respiration 29, 30. For this, we incubated the 2C::tbGFP cell line with CellROX, a ROS‐sensitive fluorescent probe which detects the oxidative species HO• and •$O_{2}^{-}$, and measured fluorescence intensity in 2‐cell‐like (2C::tbGFP^+^) and ES (2C::tbGFP^–^) cells by direct visualization using confocal microscopy (Fig 1G). CellROX staining was heterogeneous in mouse ESCs, but 2‐cell‐like cells clearly displayed an overall lower reactivity to CellROX, indicating lower ROS accumulation (Fig 1G). FACS analysis confirmed these results quantitatively, indicating reduced ROS levels in 2‐cell‐like cells (Figs 1H and EV1C), in line with the reduced respiratory activity of these cells.

**Figure 2:**
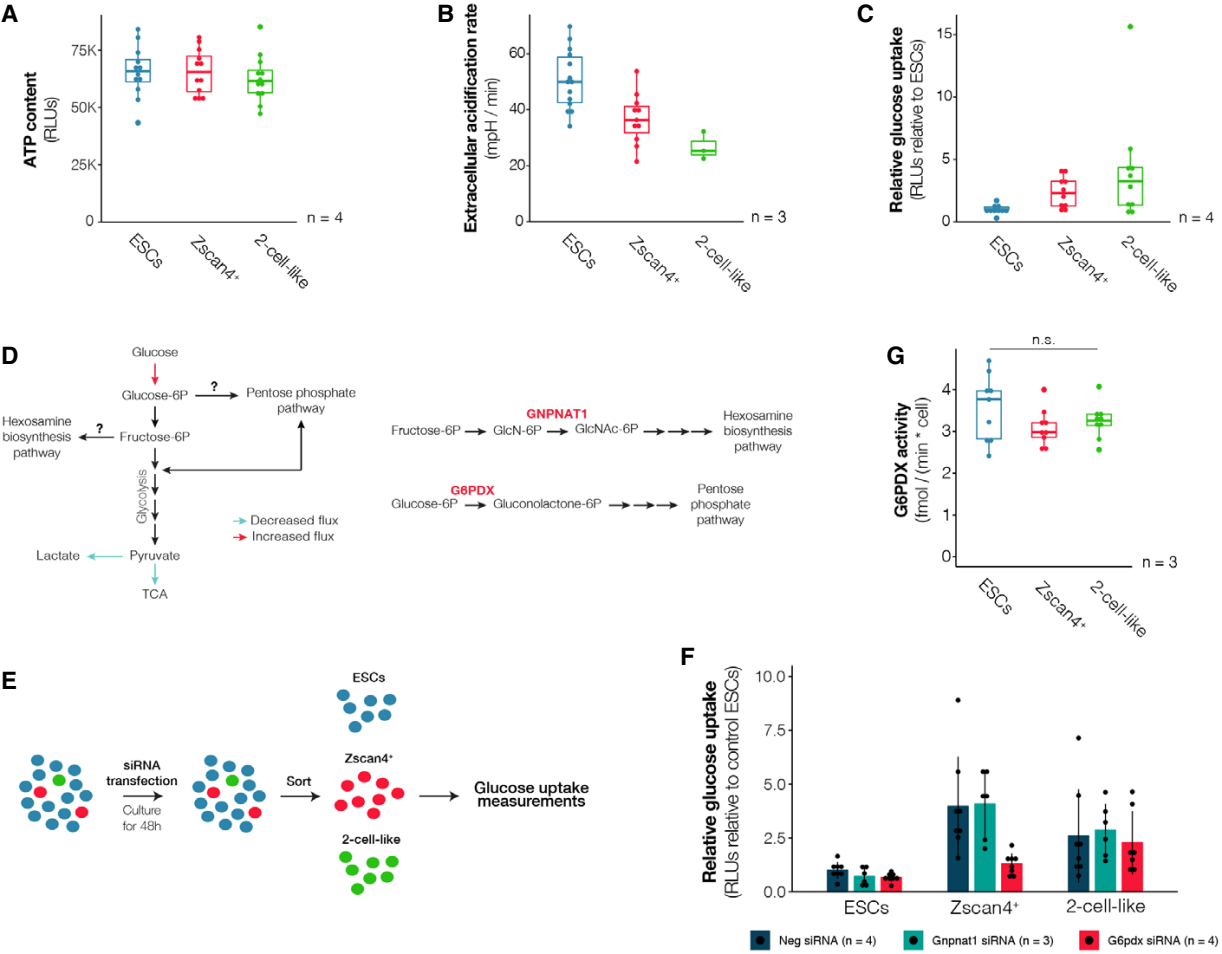
Increased glucose uptake supports higher flux into the pentose phosphate pathway in Zscan4^+^ cells ATP content in ES (blue), Zscan4^+^ (red) and 2‐cell‐like cells (green) across four independent biological replicates.Extracellular acidification rate of ES (blue), Zscan4^+^ (red) and 2‐cell‐like cells (green) across three independent biological replicates performed on the Seahorse extracellular flux analyser.Glucose uptake rates in Zscan4^+^ (red) and 2‐cell‐like cells (green) were measured using a luciferase‐based assay across four independent biological replicates and are represented relative to those of control ES cells (blue).Schematic representation of measured fluxes (left). In order to ascertain whether the increased glucose uptake observed leads to higher flux into the hexosamine biosynthesis pathway (HBP) or the pentose phosphate pathway (PPP), one enzyme of each pathway was disrupted through siRNA‐mediated knockdown (right).Experimental design. ESC cultures were transfected with siRNAs targeting Gnpnat1 (HBP), G6pdx (PPP) or a negative control siRNA (Neg). After 48 h of culture, cells were FACS‐sorted into a 96‐well plate based on their fluorescent reporters and glucose uptake rates were measured using a luciferase‐based assay.Glucose uptake rates upon knockdown of Gnpnat1 or a G6pdx were measured in ES, Zscan4^+^ and 2‐cell‐like cells. Measurements were quantified relative to the glucose uptake rate of ESCs transfected with a negative control siRNA. Shown are the mean ± s.d. of the indicated number of independent cell cultures, performed across 2 or more independent biological replicates each.Glucose‐6‐phosphate dehydrogenase activity was measured in ES (blue), Zscan4^+^ (red) and 2‐cell‐like cell (green) lysates using a fluorometric assay. Measurements were obtained from three independent biological replicates, performed in three technical replicates each. n.s.—not significant; one‐way ANOVA.Data information: In panels (A–C and G), boxes indicate the range between the first and third quartile, the band depicts the median, and the whiskers span the range of the data while extending no further than 1.5 times the interquartile range. Individual dots indicate the measurements obtained in each individual technical replicate.Source data are available online for this figure.

**Figure EV5:**
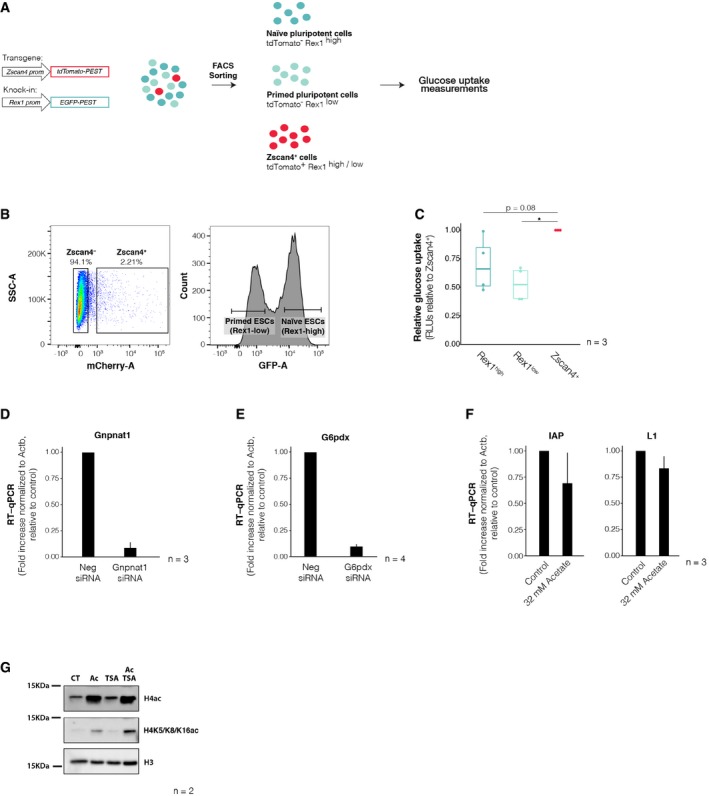
Zscan4^+^ cells exhibit higher glucose uptake than both naïve and primed ES cells AExperimental design. ES cells were cultured in serum/LIF conditions over feeders for at least 5 days in the absence of 2i, and subsequently FACS‐sorted into naïve pluripotent, primed pluripotent or Zscan4^+^ cells. Glucose uptake rates were measured thereafter. Reporter constructs employed to identify all three distinct populations are represented on the left. An EGFP reporter driven by the Rex1 endogenous promoter was used to distinguish between Rex1‐high (naïve pluripotent) and Rex1‐low (primed pluripotent) cells, and a tdTomato cassette expressed downstream of an ectopic Zscan4c promoter was used to mark Zscan4^+^ cells.BRepresentative sorting gates used for the isolation of naïve pluripotent, primed pluripotent or Zscan4^+^ cells. Zscan4^+^ cells were defined as those positive for Zscan4c::tdTomato reporter (left), irrespective of their Rex1‐EGFP fluorescence level. Naïve pluripotent and primed pluripotent stem cells were gated based on the bimodality of the Rex1‐EGFP distribution (right).CGlucose uptake rates in Zscan4^+^ cells (red), naïve pluripotent stem cells (dark blue) and primed pluripotent stem cells (light blue) were measured using a luciferase‐based assay across three independent biological replicates. Measurements are represented relative to the levels of Zscan4^+^ cells. Boxes indicate the range between the first and third quartile, the band specifies the median, and the whiskers span the range of the data while extending no further than 1.5 times the interquartile range. Individual dots indicate the measurements obtained in each technical replicate. *P < 0.05; one sample *t*‐test.D, ERT–qPCR analysis of the indicated genes after transfection with the corresponding siRNAs. Shown are mean values ± s.d. of two technical replicates from three independent biological replicates.FRT–qPCR of the indicated repeats upon 24 h of sodium acetate treatment. Shown are the mean ± s.d. of three independent cell cultures, performed in two technical replicates.GWestern blot for the indicated antibodies in lysates derived from control and acetate‐ and/or TSA‐treated cultures. Source data are available online for this figure.

Given the decreased respiration and overall lower mitochondrial activity in 2‐cell‐like cells, we next addressed whether ATP levels might be compromised in 2‐cell‐like cells. We FACS‐sorted equal numbers of ES, Zscan4^+^ and 2‐cell‐like cells and measured ATP levels using a luciferase‐based assay. Unexpectedly, we did not detect significant changes in ATP levels in any of the three cell populations (Fig 2A). Our observation that 2‐cell‐like cells display similar ATP levels to ESCs suggests that the lower respiratory activity observed in 2‐cell‐like cells is compensated by either decreased energy expenditure or increased glycolytic activity. To discern between the above possibilities, we next determined glycolytic activity in 2‐cell‐like cells by measuring the extracellular acidification rate and the glucose uptake rate of ESCs, Zscan4^+^ and 2‐cell‐like cells. Extracellular acidification is mainly the result of glycolytic activity and arises through the excretion of lactic acid, one of the major glycolytic end products, to the extracellular media. Under standard ESC culture medium conditions, Zscan4^+^ and 2‐cell‐like cells exhibited a lower extracellular acidification rate than ESCs, suggesting lower glycolytic output (Fig 2B). Surprisingly, however, glucose uptake rate measurements in all three populations indicated that Zscan4^+^ and 2‐cell‐like cells exhibited higher rates of glucose uptake than ESCs (Fig 2C), an observation at odds with the lower extracellular acidification rates measured in these two cell populations, which suggests an alternative, non‐glycolytic fate for the consumed glucose. Because Zscan4^+^ and 2‐cell‐like cells derive primarily from naïve ESCs 16, we addressed whether the observed differences in glucose uptake between ESCs and 2‐cell‐like cells are related to their preferential origin from the naïve ESC state. Mouse ESCs exist in two metastable states—naïve and primed—, which differ in their metabolic state 10. Naïve cells express high levels of the transcription factor Rex1 (*Rex1* ^*high*^ ESCs) 4, 9 and rely on a mixture of glycolytic and aerobic metabolism. In contrast, primed pluripotent stem cells express low levels of Rex1 (*Rex1* ^*low*^) and rely almost exclusively on glycolysis to satisfy their energetic demands 10. Glucose uptake tends to be higher in ESCs grown in 2i—where ESCs are primarily in a naïve, *Rex1* ^*high*^—compared to serum/LIF conditions—in which ESCs cycle between *Rex1* ^*high*^ and *Rex1* ^*low*^ pluripotency states 31. We FACS‐sorted equal numbers of Rex1^high^ ESCs, Rex1^low^ ESCs and Zscan4^+^ cells and measured glucose uptake as before using a luciferase‐based assay (Fig EV5A and B). We find that Zscan4^+^ cells exhibited higher glucose uptake than either primed or naïve cells, suggesting that the differences in glucose uptake between ESCs and early‐embryonic‐like cells are not related to their pluripotent state (Fig EV5C).

Our observations above, indicating lower mitochondrial respiration and lower lactate production in 2‐cell‐like cells, are at odds with their higher glucose uptake and suggest an alternative non‐glycolytic fate for the bulk of the consumed glucose (Fig 2C). Therefore, we asked whether 2‐cell‐like cells divert their intracellular glucose towards other pathways such as the hexosamine biosynthetic pathway (HBP) or the pentose phosphate pathway (PPP). To address this, we downregulated each of these two pathways using RNAi—in such a way that flux through the corresponding pathway would be stalled upon knockdown of the targeted enzyme (Fig 2D and E)—and measured glucose uptake 48 h later. Because of the extended culture period required to achieve an efficient knockdown (Fig EV5D and E) and in order to maintain cellular viability, we were unable to include any glycolytic enzymes as a positive control in this assay. Downregulation of *Gnpnat1*, which catalyses the transfer of an acetyl group from Ac‐CoA to glucosamine‐6‐phosphate, did not reduce glucose uptake in either Zscan4^+^ or 2‐cell‐like cells, relative to the negative control siRNA (Figs 2F and EV5D). In contrast, RNAi for *G6pdx*, which catalyses the first and rate‐limiting reaction of the oxidative branch of the pentose phosphate pathway (PPP), resulted in a decrease in glucose consumption of ESCs (−28%) and Zscan4^+^ (−65%), but barely affected the glucose uptake of 2‐cell‐like cells (−13%), which remained mostly unchanged (Figs 2F and EV5E). Importantly, we did not detect any changes in G6PDX activity in lysates from the three cell types (Fig 2G). Thus, it would seem that increased glucose uptake supports higher flux into the pentose phosphate pathway in Zscan4^+^ cells. Altogether, our results indicate that early‐embryonic‐like cells exhibit decreased glycolytic and respiratory activity, altered mitochondrial morphology and increased glucose uptake, suggesting a shift of the metabolic programme during reprogramming to the 2‐cell‐like state.

**Figure 3:**
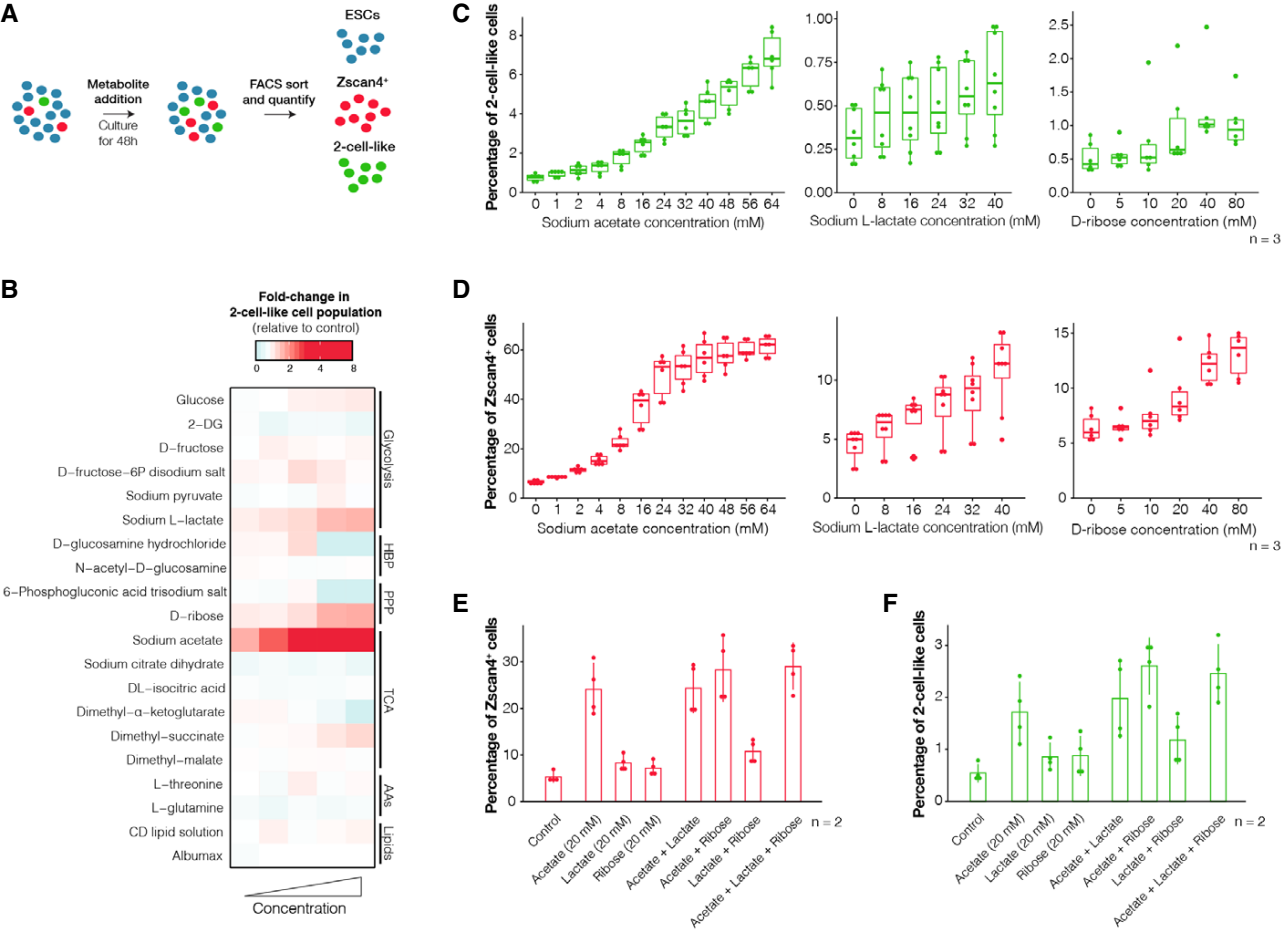
Induction of Zscan4^+^ and 2‐cell‐like cells by defined metabolites AExperimental design. ESC cultures were supplemented with increasing concentrations (see Table EV1) of 20 distinct metabolites for 48 h. The abundance of the Zscan4^+^ and 2‐cell‐like cells was quantified by FACS according to the reporters shown in Fig 1B.BHeatmap showing the effect of the 20 metabolites tested on 2‐cell‐like cell levels.C–FPercentage of Zscan4^+^ and 2‐cell‐like cells in cultures treated with increasing concentrations (C and D) or varying combinations (E and F) of sodium acetate, sodium l‐lactate or d‐ribose. Shown are the mean ± s.d. of two independent cell cultures, performed across 3 (C and D) or 2 (E and F) biological replicates. Boxes indicate the range between the first and third quartile, the band depicts the median, and the whiskers span the range of the data while extending no further than 1.5 times the interquartile range. Individual dots indicate the measurements obtained in each of the 6 (C and D) or 4 (E and F) technical replicates. Source data are available online for this figure.

Given the observed changes in metabolic activity described above, we hypothesized that the addition of specific metabolites may alter the number of early‐embryonic‐like cells present in mouse ESC cultures. Thus, we next addressed whether the number of Zscan4^+^ and 2‐cell‐like cells is affected upon addition of specific metabolites to the medium (Fig 3A). We incubated our double reporter cell line (Zscan4c::mCherry, 2C::tbGFP) with varying concentrations of 20 selected metabolites for 48 h and quantified the number of Zscan4^+^ and 2‐cell‐like cells in each of these conditions using FACS (Fig 3B, Table 4 and Table EV1). Overall, we identified three metabolites that displayed a robust induction of 2‐cell‐like cells in a dose‐dependent manner. These included sodium l‐lactate, d‐ribose and sodium acetate. Sodium acetate displayed the strongest effect in inducing the 2‐cell‐like cell population (Fig 3B), which reached up to 8% of the culture at the maximum dose applied (Fig 3C). Similarly, addition of d‐ribose or sodium l‐lactate also induced the 2‐cell‐like cell population in a dose‐dependent manner. Addition of sodium acetate, sodium l‐lactate or d‐ribose also resulted in a clear induction of Zscan4^+^ cells—which reached up to ~ 60% of the total cell population at the highest sodium acetate dose applied—suggesting that these metabolites induce *bona fide* 2‐cell‐like cells (Fig 3D). Combining d‐ribose with sodium acetate or sodium l‐lactate resulted in an increased number of Zscan4^+^ and 2‐cell‐like cells compared to sodium acetate or sodium l‐lactate alone (Fig 3E and F). However, addition of sodium acetate and sodium l‐lactate together did not cause additive effects on the number of either Zscan4^+^ or 2‐cell‐like cells (Fig 3E and F). These results suggest that sodium acetate and sodium l‐lactate may induce 2‐cell‐like cells through the same pathway.

**Figure 4:**
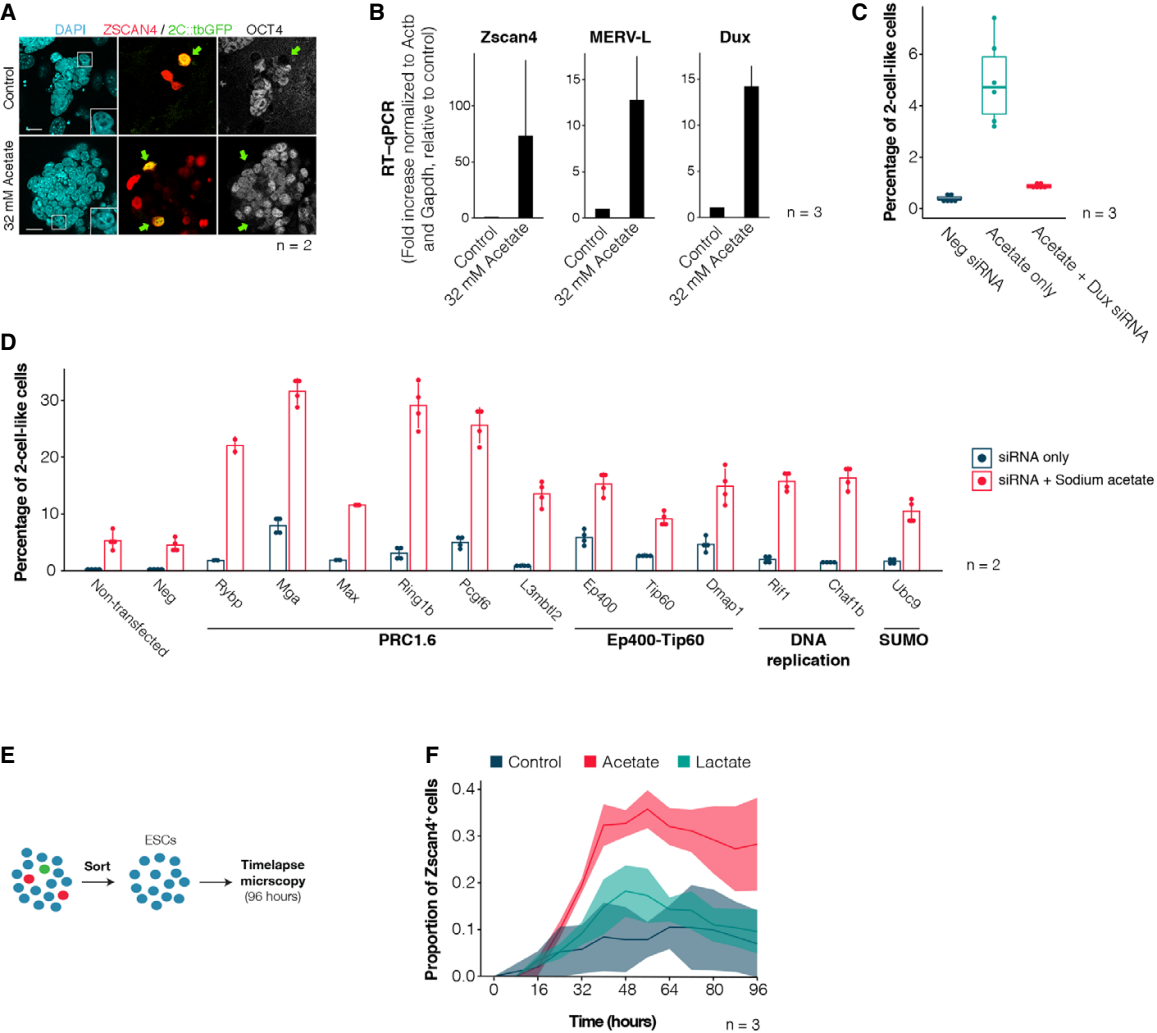
Sodium acetate induces Zscan4^+^ and 2‐cell‐like cells Immunofluorescence staining for OCT4, ZSCAN4 and 2C::tbGFP in control and acetate‐treated ESC cultures. Green arrows indicate 2‐cell‐like cells, and inlets highlight their DAPI structure. Scale bar, 20 μm.RT–qPCR of the indicated genes in ESC cultures treated with sodium acetate for 24 h. Shown are the mean ± s.d. of three independent cell cultures, performed in two technical replicates.Percentage of 2‐cell‐like cells obtained upon transfection of control or Dux‐targeting siRNAs in control conditions or in combination with sodium acetate treatment. Measurements were obtained from two independent cell cultures, performed across three independent biological replicates. Boxes indicate the range between the first and third quartile, the band depicts the median, and the whiskers span the range of the data while extending no further than 1.5 times the interquartile range. Individual dots indicate the measurements obtained in each technical replicate.Percentage of 2‐cell‐like cells obtained upon transfection siRNAs targeting the indicated chromatin factors in control conditions or in combination with sodium acetate treatment. Shown are the mean ± s.d. of four independent cell cultures, performed across two independent biological replicates.Experimental design. ESC cultures were FACS‐sorted to remove Zscan4^+^ and 2‐cell‐like cells and plated in a glass bottom 96‐well plate. Cells were then imaged for 96 h in the presence of sodium acetate (32 mM), sodium l‐lactate (32 mM) or in control conditions.Proportion of Zscan4^+^ cells at various timepoints during the time‐lapse experiment. Shown are the mean ± s.d. of three independent experiments. Source data are available online for this figure.

Metabolite‐induced 2‐cell‐like cells displayed the same molecular features of endogenous 2‐cell‐like cells, namely increased levels of ZSCAN4, loss of chromocentres and loss of OCT4 protein (Fig 4A). In addition, sodium acetate treatment induced a robust increase in *Zscan4* and MERVL transcripts (Fig 4B), although levels of L1 and IAP remained unchanged (Fig EV5F), consistent with the known transcriptional features of 2‐cell‐like cells 11, 15. The transcription factor DUX, which has been recently shown to bind to and regulate MERVL expression 12, 22, 32, was also upregulated upon acetate treatment (Fig 4B), and induction of 2‐cell‐like cells by acetate was significantly reduced upon Dux siRNA transfection (Fig 4C). In addition, acetate incubation led to a synergistic effect in the induction of 2‐cell‐like cells, when combined with siRNA for specific chromatin modifiers known to induce 2‐cell‐like cells (Fig 4D) 16. This suggests that sodium acetate induces 2‐cell‐like cells through parallel pathways to those of the chromatin modifiers tested. The induction of 2‐cell‐like cells by acetate is in line with the known increase in levels of histone acetylation in 2‐cell‐like cells 11, 15 and suggests that at least part of the effect observed upon acetate supplementation might be linked to increased levels of histone acetylation. Indeed, we find that acetate supplementation led to increased global levels of histone acetylation (Fig EV5G), consistent with previous reports 33. We also addressed whether sodium acetate and l‐lactate increase the 2‐cell‐like cell population by promoting either maintenance or induction using time‐lapse microscopy with a Zscan4 reporter 16 after removal of Zscan4^+^ and 2‐cell‐like cells. Our results suggest that both sodium acetate and l‐lactate induce rather than stabilize Zscan4^+^ cells (Fig 4E and F). Further studies are needed to determine the mechanism through which these metabolites induce 2‐cell‐like cells, which may encompass metabolic as well as epigenetic mechanisms. Lactate, for example, may act through glycolytic metabolism, but potentially also through the inhibition of HDACs 34. Thus, we conclude that 2‐cell‐like cells display a global metabolic shift compared to ES cells and that specific metabolites can induce the emergence of 2‐cell‐like cells in culture.

**Figure 5:**
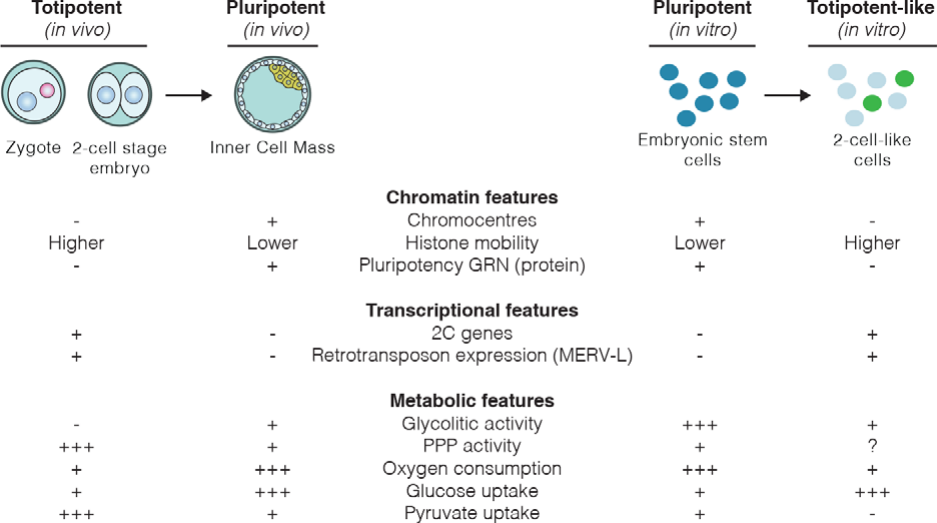
Summary of known molecular features of totipotent and totipotent‐like cells Overview of the known molecular characteristics of totipotent and pluripotent cells *in vivo* and *in vitro*, now including metabolic features.

Overall, our work shows that early‐embryonic‐like cells (Zscan4^+^ and 2‐cell‐like cells) differ in their metabolic activity from ESCs. Similarly to the 2‐cell‐stage embryo 25, 28, 35, 36, 37, 2‐cell‐like cells seem to exhibit a “quiet” metabolism, characterized by low glycolytic and respiratory activity, as well as altered mitochondrial morphology and lower ROS production (Fig 5). However, our results also show that some differences between early‐embryonic‐like cells and early embryos exist, most notably in terms of their substrate uptake rates. Intriguingly, we observed that early‐embryonic‐like cells are characterized by higher glucose uptake rates than ESCs and the inability to increase respiration using exogenous pyruvate. It is unclear to what extent such differences between early embryos and early‐embryonic‐like cells reflect differences in culture conditions or a fundamental difference in metabolic requirements between a transient totipotent embryo and the self‐renewing pluripotent state from which early‐embryonic‐like cells arise.

Morula and blastocyst‐stage embryos rely on glucose to produce energy through a combination of glycolysis and oxidative phosphorylation, while cleavage embryos up to the 8‐cell stage rely exclusively on monocarboxylates such as pyruvate and lactate 23. These changes in substrate requirements have been proposed to reflect the embryo's need to provide sufficient supplies of specific metabolites—such as acetyl‐CoA or α‐ketoglutarate—that are required for the activation of the embryonic genome 38, while maintaining an overall low metabolic activity in order to restrict ROS production and oxidative damage 35. Intriguingly, early‐embryonic‐like cells upregulate glucose uptake although without the consequent, expected increase in lactate production, suggesting alternative fates for the extra glucose consumed. PPP disruption led to a significant decrease in glucose consumption in Zscan4^+^ cells, but not in 2‐cell‐like cells, suggesting high glucose flux into this pathway in Zscan4^+^ cells. Other pathways might be active in 2‐cell‐like cells and may contribute to the high glucose consumption seen in 2‐cell‐like cells. These may include respiration‐uncoupled pyruvate metabolism into acetyl‐CoA or PPP activity through the non‐oxidative branch. The former is supported by the fact that acetate strongly increases 2‐cell‐like cells in culture, which display higher levels of global histone acetylation compared to ESCs 11, 15.

## Materials and Methods

### Cell culture

All cell lines used in this study, unless otherwise stated, were grown in media containing DMEM‐Glutamax‐I, 15% foetal calf serum, 2× LIF, 2‐betamercaptoethanol, non‐essential amino acids, penicillin and streptomycin on feeders. Medium supplemented with 2i (3 μM CHIR99021 and 1 μM PD0325901, Miltenyi Biotec) was used for the establishment of stable cell lines and for their expansion and maintenance. After removal of 2i, cells were cultured for at least 5 days in serum/LIF conditions on feeder cells before being used for experiments, unless otherwise stated. Lipofectamine RNAi MAX (Life Technologies) was used for siRNA transfection.

### Reporter cell lines

Six different reporter ES cell lines derived from the E14 cell line were used in this study and are described in detail in Tables 1 and 2. 2C‐reporter cell lines harbouring either a tdTomato, an EGFP or a turboGFP cassette were described previously 15. A 2C::turboGFP reporter cell line with constitutive tdTomato expression driven by the CAG promoter was used for the electron microscopy experiments and is described in further detail in 16. All measurements on the Zscan4^+^ and 2‐cell‐like populations were performed on a triple reporter cell line carrying 2C::tbGFP, Zscan4c::mCherry and constitutive H2B‐iRFP constructs. This cell line incorporates a Zscan4 reporter construct (kindly provided by M. Ko) and is described elsewhere 16. The Rex1 reporter cell line used for the glucose uptake measurements on naïve and primed pluripotent ES cells was kindly provided by A. Smith 4, and the generation of the Rex1 reporter line with the Zscan4 reporter has been described previously 16.

**Table 2:** Reporter cell lines used in each experiment

Experiment	Cell line
Seahorse extracellular flux assay	tbg4ZH
ATP content measurements	tbg4ZH
Glucose uptake measurements	tbg4ZH
Metabolite incubations	tbg4ZH and tbg4‐12
ROS measurements	tbg4
Immunofluorescence experiments	tbg4
Electron microscopy	tbg4‐12
RT–qPCR on control and acetate‐treated cells	2C‐EGFP
Glucose uptake measurements on naïve, primed and Zscan4 cells	Rex1‐Zscan4
G6PDH activity assay	tbg4ZH
Histone acetylation western blot	tbg4
Autophagy measurements	2C‐tdTomato
Time‐lapse experiments	tbg4ZH
Chromatin factor knockdowns	2C‐EGFP

### Fluorescence‐assisted cell sorting

Cells were washed with room temperature sterile PBS, trypsinized and resuspended in ice‐cold sterile 0.5% BSA PBS solution. Sorting was performed using a BD BioSciences FACS Aria II or III. During sorting, cells were collected in culture medium and kept at 4°C during the sort. Analysis of FACS data was performed using the FlowJo software.

### Measurements of cellular oxygen consumption rates and extracellular acidification rates

To measure the oxygen consumption and extracellular acidification rates, a Seahorse XFe96 Flux Analyser was used. Five × 10^4^ cells were FACS‐sorted, centrifuged and seeded in XF96 plates 3–4 h prior to the assay in standard ESC medium as described above. Prior to the assay, culture medium was gently replaced with Seahorse assay medium containing glucose (25 mM), l‐glutamine (2 mM) and sodium pyruvate (1 mM). A gentle wash using Seahorse medium was performed to minimize carry‐over of the DMEM medium. Basal, maximal (FCCP, 250 nM) and non‐mitochondrial respiration (rotenone/antimycin A, 500 nM) as well as extracellular acidification were determined over 3 min of measurement. Basal respiratory capacity results shown derive from three independent biological replicates. Maximal and non‐mitochondrial respiratory capacity results shown derive from two independent biological replicates.

### Measurements of cellular oxygen consumption rates upon lactate or pyruvate supplementation

To measure the oxygen consumption rate upon acute sodium lactate or sodium pyruvate supplementation, a Seahorse XFe96 Flux Analyser was used. Five × 10^4^ cells were FACS‐sorted, centrifuged and seeded in XF96 plates 3–4 h prior to the assay in standard ES cell medium as described above. Before the assay, the ES culture medium was gently replaced with glucose‐free Seahorse assay medium containing solely l‐glutamine (2 mM). A gentle wash using Seahorse medium was performed to minimize carry‐over of the DMEM medium. Basal, pyruvate or lactate‐stimulated (20 mM), maximal (FCCP, 250 nM) and non‐mitochondrial respiration (rotenone/antimycin A, 500 nM) was determined over 3 min of measurement. Respiratory rate measurements for each metabolite derive from three independent biological replicates.

### ROS measurements

Cells were plated over gelatin‐coated cell culture dishes and treated with CellROX Deep Red reagent (ThermoFisher Scientific) diluted in culture medium at a final concentration of 5 μM for 30 min. For image acquisition, cells were washed three times with PBS, stained with Hoechst 33342 diluted in culture medium and imaged using a 60× 1.4 NA Plan‐Apochromat VC objective on a Nikon Ti‐E equipped with a Yokogawa CSU‐X1 spinning disc head and a Photometrics Evolve 512 EMCCD camera. For flow cytometry analysis, cells were washed three times in PBS, trypsinized, resuspended in 0.5% BSA PBS and analysed using a FACS Aria II.

### ATP content measurements

ATP content was measured using the luciferase‐based CellTiter‐Glo assay (Promega) according to the manufacturer's instructions with a few modifications in order to couple it to FACS sorting. Briefly, 1,000 ES cells, Zscan4^+^ cells or 2‐cell‐like cells were FACS‐sorted in biological triplicates into 100 μl of sterile PBS deposited on the wells of a white 96‐well plate. Following addition of 100 μl of CellTiter‐Glo reagent, plates were shaken for 2 min at room temperature and luminescence was allowed to stabilize for 10 min. Finally, luminescence was measured on an Orion II microplate luminometer (Berthold Titertek) with 1 s integration time. Readings from negative control wells where no cells were sorted were subtracted from all other measurements to account for background.

### Glucose uptake measurements

Glucose uptake rates were measured using the luciferase‐based Glucose Uptake‐Glo assay (Promega) according to the manufacturer's instructions with a few modifications in order to couple it to FACS sorting. Briefly, 2,500 ES cells, Zscan4^+^ cells or 2‐cell‐like cells were FACS‐sorted in biological triplicates into 20 μl of sterile PBS deposited on the wells of a white 96‐well plate (for a total volume of 35 μl). Following addition of 15 μl of 3.33 mM 2‐DG diluted in PBS (for a final concentration of 1 mM), plates were shaken for 1 min and incubated at room temperature for 20 min. Following addition of stop, neutralization and luciferase solution according to manufacturer's instructions, luminescence was measured on an Orion II microplate luminometer (Berthold Titertek) luminometer with 1 s integration time. Readings from negative control wells to which 2,500 ES cells were sorted but no 2‐DG was added were subtracted from all other measurements to account for background. In the case of the glucose uptake measurements performed on naïve, primed and Zscan4^+^ cells, experiments were carried as described above but sorting was performed on Rex1‐high and Rex1‐low populations instead.

### Glucose uptake measurements after Gnpnat1 or G6pdx knockdown

Forty‐eight hours before measurements, 0.375 × 10^6^ ES cells were transfected with siRNAs targeting Gnpnat1, G6pdx or a non‐targeting siRNA in biological triplicates using Lipofectamine RNAi MAX (ThermoFisher Scientific). Cell culture medium was replaced after 24 h. Following this time period, cells were washed with room temperature sterile PBS, trypsinized, resuspended in ice‐cold sterile 0.5% BSA PBS solution and FACS‐sorted as described above for the rest of the glucose uptake measurements. All three siRNA‐transfected cultures were sorted into the same plate in biological duplicates and were assayed simultaneously. Knockdown efficiency was determined as described below.

### Real‐time RT–qPCR

Total RNA was extracted from ES cells using the ReliaPrep miniprep kit (Promega), and reverse transcription was performed with SuperScript II (ThermoFisher Scientific) with oligodT oligos. For the MERVL and Zscan4 RT–qPCR analysis, total RNA was extracted from ES cells with the RNeasy Plus mini kit (Qiagen) and treated with turbo DNase (ThermoFisher Scientific) to remove genomic DNA. Reverse transcription was performed with SuperScript II (ThermoFisher Scientific) with random hexamers. Real‐time PCR was performed with Lightcycler 480 SYBR Green I Master Mix (Roche) on a LightCycler 96 Real‐time PCR System (Roche). The relative expression level was normalized to *Gapdh* and *Actb* (for MERVL, Zscan4, Dux, IAP and L1), and to Actb only (for RT–qPCR analysis of siRNA efficiency). Primers used in this study are described in Table 3.

**Table 3:** Oligonucleotide sequences used in this study

Gene	Forward primer	Reverse primer	siRNA target sequence
Actb	CACGGTTGGCCTTAGGGTTCAG	GCTGTATTCCCCTCCATCGTG	
Gapdh	GCCTGCTTCACCACCTTCTT	CATGGCCTTCCGTGTTCCTA	
Zscan4	GCTGTTGTTTCAAAAGCTTGATGACTTC	GAGATTCATGGAGAGTCTGACTGATGAGTG	
MERVL	GAGGCTCCAAACAGCATCTCTA	CTCTACCACTTGGACCATATGAC	
IAP	AAGCAGCAATCACCCACTTTGG	CAATCATTAGATGCGGCTGCCAAG	
L1	GGACCAGAAAAGAAATTCCTCCCG	CTCTTCTGGCTTTCATAGTCTCTGG	
Dux	AGGCCCTGCTATCAACTTTCA	CTCCTCTCCACTGCGATTCC	
Gnpnat1	CGCTCCAGTGCGACTTTA	TGGGCTGCAGCAACAAAAAT	GGCAAACUGUUAUUAUCAA
G6pdx	GATCATCAGCGATGTTATGC	CTCTGAGATACACTTCAACAC	GAGGAGUUCUUUGCCCGUA
Non‐targeting siRNA #1			UAGCGACUAAACACAUCAA
Ubc9			AGAUCUAAGUCGCUCCGUA

### Metabolite incubations

For the metabolite incubation experiments, solid metabolites were diluted in PBS to generate a high concentration stock solution, and liquid metabolites were added directly to the culture media. Detailed references for all the metabolites used are listed in Table 4. Various volumes of these stock solutions were then added to ES cell cultures at the concentrations described in Table EV1. ES cells were grown in 24‐well plates for 48 h over feeders, and the media were replaced daily, using metabolite‐supplemented media. Following this culture period, cells were trypsinized and FACS‐sorted for quantification.

**Table 4:** Metabolites used in this study

Compound name	Reference
l‐threonine	T8441‐25G
Sodium citrate dihydrate	W302600‐1KG
Dimethyl‐(S)‐(−)‐malate	374318‐5G
Dimethyl‐succinate	W239607
Dimethyl‐a‐ketoglutarate	349631‐5G
Monomethyl fumarate	A651419‐10G
l‐glutamine	G‐3126‐100G
*N*‐acetyl‐d‐glucosamine	A3286‐5G
D‐fructose 6‐phosphate disodium salt	F3627‐500mg
Sodium l‐lactate	L7022
DL‐isocitric acid	I1252‐1G
Sodium pyruvate	P2256‐5G
Sodium acetate	S5636‐250G
Nicotinamide mononucleotide	N0636‐100G
Albumax I Lipid‐Rich BSA	11020021
D‐(−)‐ribose	R7500‐5G
6‐phosphogluconic acid trisodium salt	P7877‐100mg
D‐(+)‐glucosamine hydrochloride	G4875‐25
D‐(−)‐fructose	F0127‐100G
D‐(+)‐glucose	G8270‐1KG
Chemically defined lipid concentrate	11905031
UK‐5099	PZ0160‐5mg
Sodium oxamate	02751‐5g
2‐Deoxy‐D‐glucose	D8375‐1g

### Immunofluorescence

Cells were cultured over feeder‐coated coverslips, fixed in 4% PFA for 10 min at room temperature and permeabilized with 0.2% Triton X‐100 for another 10 min. A 3% BSA PBS blocking solution was used for blocking for 1 h. Primary antibodies were incubated overnight in blocking solution and were followed by three washes in PBS. Secondary antibodies were incubated for 1 h. Mounting was done in VECTASHIELD Hardset Mounting Medium (Vector Labs). Image acquisition was performed using a Leica SP8 confocal microscope.

### Antibodies

Antibodies used in this work were the following: mouse turboGFP (OTI2H8, Origene), rabbit Zscan4 (AB4340, EMD Millipore), panH4ac (3HH4 2C2, ThermoFisher), H4K5/K8/K16ac (in‐house produced at IGBMC), H3 (ab1791, Abcam) and goat Oct4 (sc‐8628, Santa Cruz).

### Electron microscopy

Embryos at the zygote (~ 16 hpc, *n* = 4), 2‐cell (~ 30 hpc, *n* = 5) and 8‐cell (~ 54 hpc, *n* = 4) stages were collected after natural matings of B6CBAF1/J mice, fixed in 2% formaldehyde + 2.5% glutaraldehyde in 0.1 M cacodylate buffer for 2 h at 37°C, post‐fixed 1 h at 4°C in 1% osmium tetroxide and *en bloc* stained with 1% uranyl acetate for 1 h at 4°C. Samples were then dehydrated in graded ethanol solutions (50, 70, 90, 100%) and then infiltrated with epoxy resin by a graded series of dilutions (30, 70, 100%). Due to the size of the embryos, they were flat embedded in a sandwich of Aclar (200 μm) in order to be observed using binoculars. Ultrathin sections (70 nm) were performed using an ultracut UCT ultramicrotome (Leica Microsystems, Vienna, Austria) and mounted on pioloform‐coated slot grids to avoid crossing mesh in the nucleus. They were then stained for 20 min with uranyl acetate and 5 min with lead citrate and observed with a transmission electron microscope (CM12, Philips; FEI Electron Optics, Eindhoven, the Netherlands) operated at 80 kV. Images were acquired using an Orius 1000 CCD camera (Gatan, Pleasanton, CA). ESCs (*n* = 49 sections) and 2‐cell‐like cells (*n* = 57 sections) were sorted based on 2C::turboGFP fluorescence using a FACS Aria II and then cultured for 3 h at 37°C before fixing with 2% formaldehyde + 2.5% glutaraldehyde in 0.1 M cacodylate buffer for 2 h at 37°C and treated as described above for embryos. Following acquisition, images were corrected for illumination bias using an automatic method based on intensity gradients and a bivariate polynomial modelling as previously 14 and processed using unsharp masking in Fiji.

### RNA‐seq data analysis

RNA‐seq data for 2‐cell‐like cells (2C::GFP^+^) and ESCs (2C::GFP^−^) were generated and described previously 15. Heatmaps were generated using DESeq2‐derived fold‐changes between ES cells and 2‐cell‐like cells.

### Knockdown of chromatin factors

Two days before transfection, cells were plated in gelatin‐coated dishes. The 2i inhibitors were removed from the medium 1 day before transfection. Lipofectamine RNAi MAX (Life Technologies) was used for siRNA transfection according to the manufacturer's instructions. A total of 75,000 cells were reverse‐transfected in 24‐well‐gelatin‐coated plates using 30 nM siRNA final concentration (the siRNAs employed are listed in Table 5). We used Silenced Negative Control No.1 siRNA (Life Technologies) as a negative control for siRNA treatment. The effect of RNAi was examined 2 days after transfection. Sodium acetate was applied 24 h before measurements at a concentration of 32 mM.

**Table 5:** siRNAs used in this study

Gene	Provider	Reference
Rybp	GE Healthcare	D‐042769‐01
Mga	GE Healthcare	D‐045405‐01
Max	GE Healthcare	D‐047274‐03
Ring1b	GE Healthcare	D‐042180‐01
Pcgf6	GE Healthcare	D‐049359‐01
L3mbtl2	GE Healthcare	D‐065321‐01
Ep400	GE Healthcare	D‐058750‐01
Tip60	GE Healthcare	D‐057795‐17
Dmap1	GE Healthcare	D‐059463‐02
Rif1	GE Healthcare	D‐040028‐01
Chaf1b/p60	Life Technologies	s99864

### Autophagy measurements

Measurement of autophagic activity was carried out using the CYTO‐ID autophagy detection kit (ENZO Life Sciences) according to the manufacturer's instructions. Briefly, one 6‐well‐plate well of 2C::tdTomato ES cells was trypsinized, centrifuged and washed in PBS once. Following centrifugation, cells were resuspended in 250 μl assay buffer, and afterwards, 250 μl of staining solution (1 μl Cyto‐ID dye per ml of assay buffer) was added. Cells were then incubated for 30 min at 37°C, washed once in assay buffer and resuspended in 500 μl assay buffer. Measurements were performed on a FACS Aria III. Chloroquine‐ and/or rapamycin‐treated cells were used as positive controls and exhibited a stronger fluorescence intensity, as expected. For these experiments, chloroquine and rapamycin were diluted in the culture media at 10 μM for 5–7 h and at 500 nM for 24 h, respectively.

### G6pdh activity measurements

Measurement of glucose‐6‐phosphate dehydrogenase activity was carried out using the PicoProbe Glucose‐6‐Phosphate Dehydrogenase Activity fluorometric assay kit (BioVision) according to the manufacturer's instructions with a few modifications in order to couple it to FACS sorting. Briefly, 2,500 ES cells, Zscan4^+^ cells or 2‐cell‐like cells were FACS‐sorted in biological triplicates into 35 μl of assay buffer deposited on the wells of a white 96‐well plate (for a total volume of 50 μl). An additional three wells containing ESCs were FACS‐sorted to serve as background wells. The plate was then placed on ice while positive controls, reaction mix and background mix were prepared. Lastly, 50 μl of the appropriate reaction or background mix was added to each well and measurements were performed immediately for 1 h using a CLARIOstar (BMG Labtech) fluorescent plate reader in kinetic mode at 37°C.

### Time‐lapse experiments

Prior to time‐lapse analysis, 3000 ESCs (Zscan4^−^ and 2C::tbGFP^−^) were FACS‐sorted into individual wells of a gelatin‐coated glass bottom 96‐well plate (ThermoFisher) containing 50 μl of ES cell media. Afterwards, ES cell media containing sodium acetate, sodium lactate or no added metabolites were added to each well, to a final concentration of 32 mM and a final volume of 150 μl. Cells were then allowed to attach for a couple hours. Image acquisition was carried out in four positions within each well with a 20 × 0.75 NA Plan‐Apochromat objective lens every 30 min for 96 h using a Nikon Ti‐E system equipped with the Bruker Opterra II multipoint confocal system. Images were recorded on an EMCCD camera using emission filters for turboGFP (BP520/40), mCherry (570LP) and iRFP (655LP) mounted on a FLI filter wheel. Spontaneously arising Zscan4^+^ or 2‐cell‐like cells were manually identified using the ImageJ software and quantified relative to the total number of cells present in the field of view at each specific timepoint. Time‐lapse experiments were carried out in three independent biological replicates.

### JC‐1 immunostaining

Animal experiments were carried out in compliance with local regulations (Government of Upper Bavaria). Embryos were collected from 5‐ to 8‐week‐old F1 (C57BL/6J × CBA/H) super‐ovulated females crossed with F1 males. Superovulation was induced by intraperitoneal injection of pregnant mare serum gonadotropin (PMSG, Intervet, 5 IU) and human chorionic gonadotropin (hCG, Intervet, 7.5 IU) 46–48 h later. Embryos were collected at the following times after human chorionic gonadotrophin injection (phCG): 2‐cell stage (41–43 h) and blastocyst stage (89–91 h). Embryos were randomly allocated to experimental groups, incubated for 30 min at 37°C with a 1 μM JC‐1 solution (Abcam, ab113850) or with the dilution buffer as control and then imaged in dilution buffer. Confocal microscopy was performed on a 40× oil objective on a TCS SP8 inverted confocal microscope (Leica). We used an excitation wavelength of 475 nm and emission wavelengths of 530 ± 15 nm (monomer JC‐1) and 590 ± 17.5 nm (aggregate JC‐1). Z‐sections were taken every 5 μm. Image analysis was performed using the software Fiji. For each image, the sum of slices z‐projection was performed in order to obtain the fluorescence intensity from the whole embryo. The embryo was manually segmented, and the mean intensity per embryo was calculated for both channels. The mean value for the control embryos was subtracted from experimental values for each biological replicate. The aggregate to monomeric ratio for each embryo was then calculated.

## Author contributions

DR‐T, GH, AE, MG, AI and XG performed and designed experiments. DR‐T, GH and M‐ET‐P conceived the project. DR‐T and M‐ET‐P wrote the manuscript. M‐ET‐P supervised the work.

## Conflict of interest

The authors declare that they have no conflict of interest.

## Supporting information



Expanded View Figures PDF

Table EV1

Source Data for Expanded View

Review Process File

Source Data for Figure 1

Source Data for Figure 2

Source Data for Figure 3

Source Data for Figure 4
