# Supplementary material for: A distinct metabolic state arises during the emergence of 2‐cell‐like cells
Source: EMBO Rep. 2019 Dec 18;21(1):e48354. doi: 10.15252/embr.201948354 (PMC6944916; doi:10.15252/embr.201948354)
Supplement: Supplementary file 1 — Expanded View Figures PDF [file EMBR-21-e48354-s001.pdf]

Expanded View Figures

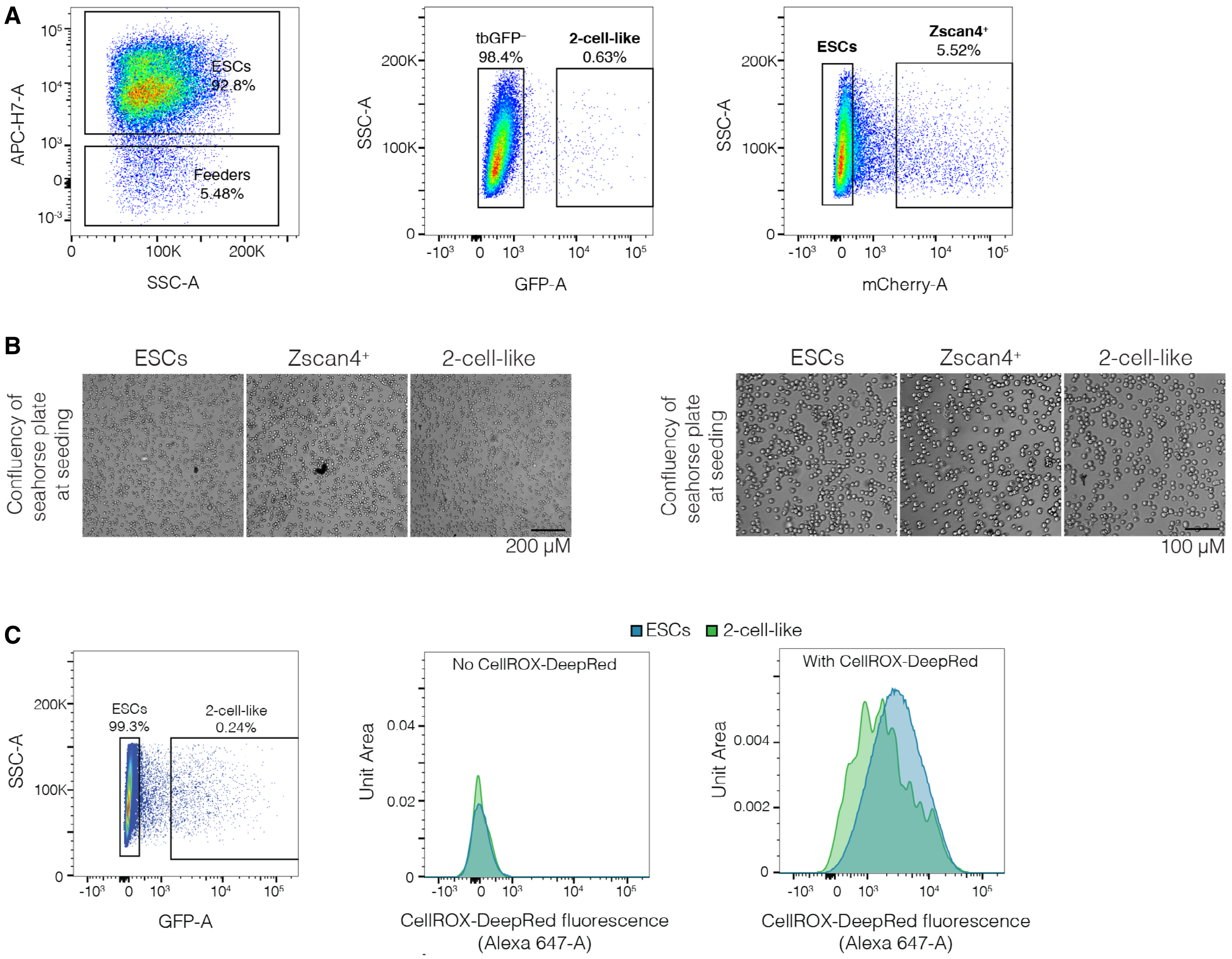

Figure EV1. Controls and set-up for Seahorse and ROS measurements.

A Representative sorting gates used for the isolation of ES, Zscan4^{+} and 2-cell-like cells used throughout this study. Feeder cells were removed on the basis of their lack of far-red fluorescence, which is higher in ES cells because of the presence of an H2B-iRFP cassette. ESCs were defined as double negative for both the Zscan4 (Zscan4c::mCherry^{-}) and the MERV-L reporters (2C::tbGFP^{-}). Zscan4^{+} cells were defined as positive for the Zscan4 reporter but negative for the MERV-L reporter, and 2-cell-like cells were defined as positive for both reporters.

B Brightfield microscopy images indicating the confluency of the three populations shortly after plating in the Seahorse extracellular flux analyser plates. Representative images, in two magnifications, for the three independent biological replicates presented in Fig 1C and D are shown.

C Representative sorting gate for ES and 2-cell-like cells (left) used for the FACS-assisted ROS measurements (right). Fluorescence intensity distributions for ES cells (blue) and 2-cell-like cells (green) in control (centre) and CellROX-treated samples (right) are shown.

© 2019 The Authors

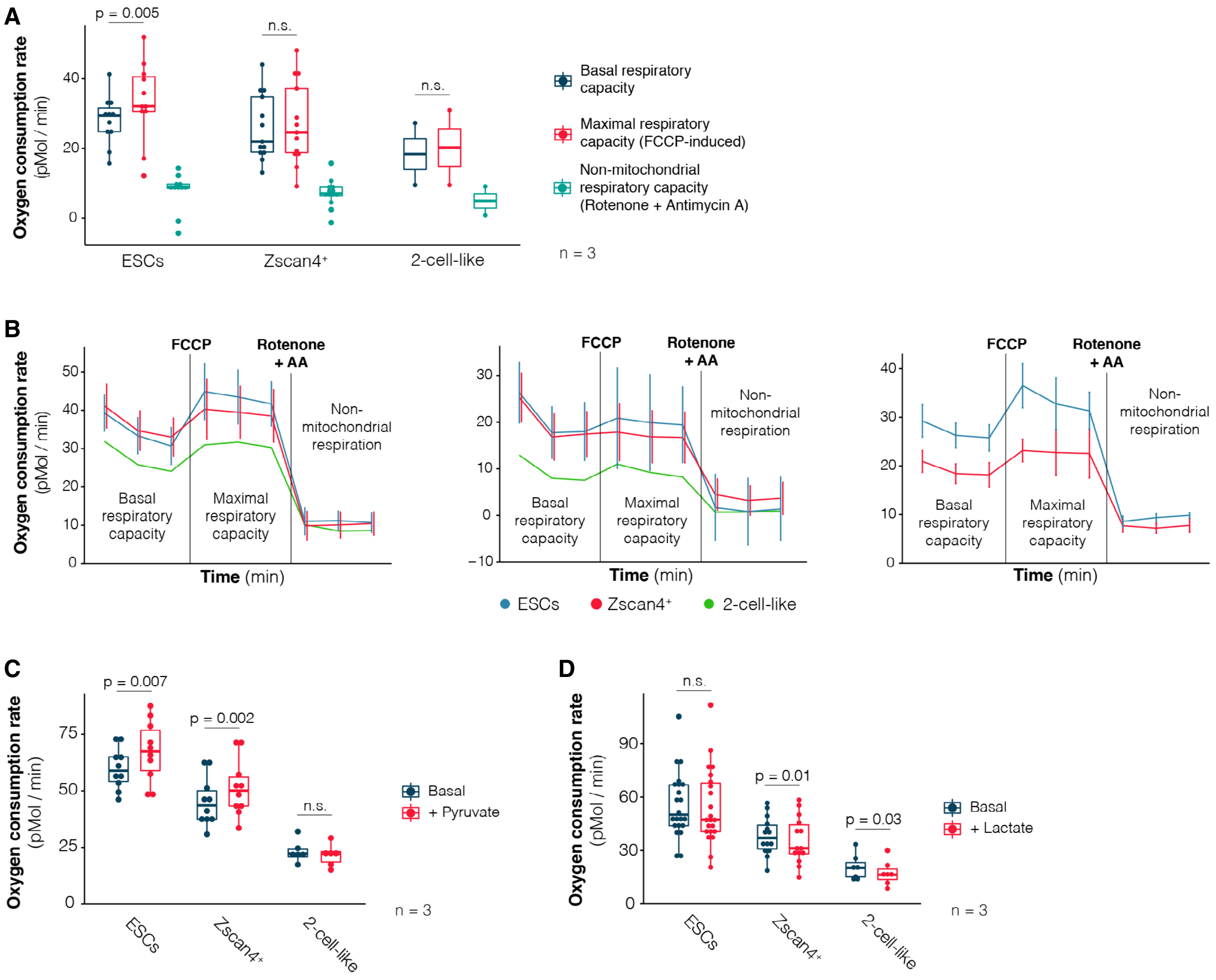

Figure EV2. Seahorse oxygen consumption rate measurements.

A Basal, maximal (FCCP-induced) and non-mitochondrial (rotenone + antimycin A-induced) oxygen consumption rate measurements of ES, Zscan4$^+$ and 2-cell-like cells performed on the Seahorse extracellular flux analyser. Assay medium was formulated to recapitulate standard ES cell culture conditions and contained glucose, L-glutamine and pyruvate. Measurements were carried out in three independent biological replicates. Note that 2-cell-like cells could only be profiled in two of those replicates. *P*-value corresponds to a paired *t*-test.

B Additional individual replicates of the oxygen consumption rate measurements of ESCs (blue line), Zscan4$^+$ (red line) and 2-cell-like cells (green line) measured on the Seahorse extracellular flux analyser, as in Fig 1C. Basal, maximal (FCCP-induced) and non-mitochondrial (rotenone-mediated) respiratory rates are indicated. Due to the low number of 2-cell-like cells in ESC cultures compared to ES and Zscan4$^+$ cells, one technical replicate of the former was analysed per biological replicate, while three technical replicates were performed for the two other populations. Accordingly, mean ± s.d. of technical replicates is shown for ES and Zscan4$^+$ cells.

C, D Oxygen consumption rate measurements of ES, Zscan4$^+$ and 2-cell-like cells performed on the Seahorse extracellular flux analyser. Basal measurements in glucose-free media and upon acute injection of sodium pyruvate (C, 20 mM) or sodium L-lactate (D, 20 mM) are shown. Note that L-glutamine—but not glucose or pyruvate—was initially present in the assay medium. Measurements from three independent biological replicates are shown. *P*-values correspond to paired *t*-tests.

Data information: Boxes indicate the range between the first and third quartile, the band depicts the median, and the whiskers span the range of the data while extending no further than 1.5 times the interquartile range. Individual dots indicate the measurements obtained in each technical replicate.
Source data are available online for this figure.

 © 2019 The Authors

Figure EV3. Electron micrographs of mitochondria in ES cells and 2-cell-like cells.

A, B Representative electron micrographs of mitochondria from ES and 2-cell-like cells. Scale bars, 1 μm.

C Autophagic flux measurements were carried out using Cyto-ID. Non-stained cells were employed as a negative control and show a distinct fluorescent profile. Chloroquine- and/or rapamycin-treated cells were used as a positive control and exhibited increased fluorescence intensity.

D FACS-assisted measurement of Cyto-ID fluorescence intensity in ES (blue) and 2-cell-like cells (green).

E Quantification of Cyto-ID fluorescence intensity in ES (blue) and 2-cell-like (green) cells. Boxes indicate the range between the first and third quartile, the band indicates the median, and the whiskers span the range of the data while extending no further than 1.5 times the interquartile range. Individual dots indicate the median fluorescence intensity measurements obtained in each biological replicate. *P*-values were calculated using a paired *t*-test.

Source data are available online for this figure.

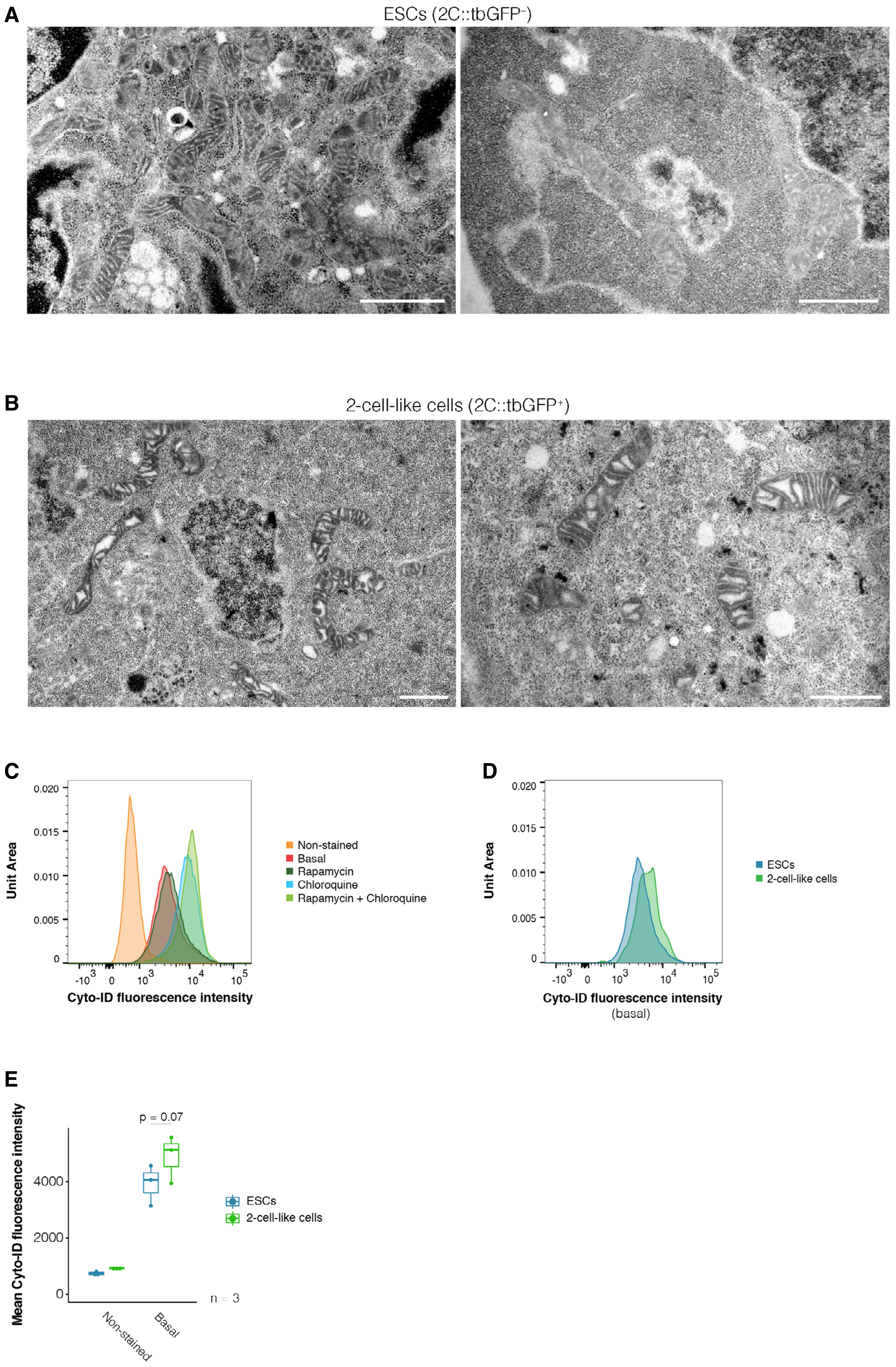

Figure EV3.

 © 2019 The Authors

Figure EV4. Electron micrographs of mitochondria from pre-implantation embryos.

A–C Representative electron micrographs of mitochondria from PN3 stage zygotes and 2-cell- and 8-cell-stage blastomeres. Scale bars, 1 μm.

D Live-cell imaging of mouse 2-cell-stage embryos and blastocysts stained with the mitochondrial membrane potential probe JC-1.

E Quantification of the ratio between aggregate and monomer emission in mouse 2-cell-stage embryos and blastocysts. Boxes indicate the range between the first and third quartile, the band specifies the median, and the whiskers span the range of the data while extending no further than 1.5 times the interquartile range. Measurements were obtained in three independent biological replicates, and each dot represents one individual embryo (n = 34, 2-cell stage; n = 16, blastocyst). n.s.—non-significant; t-test.

© 2019 The Authors

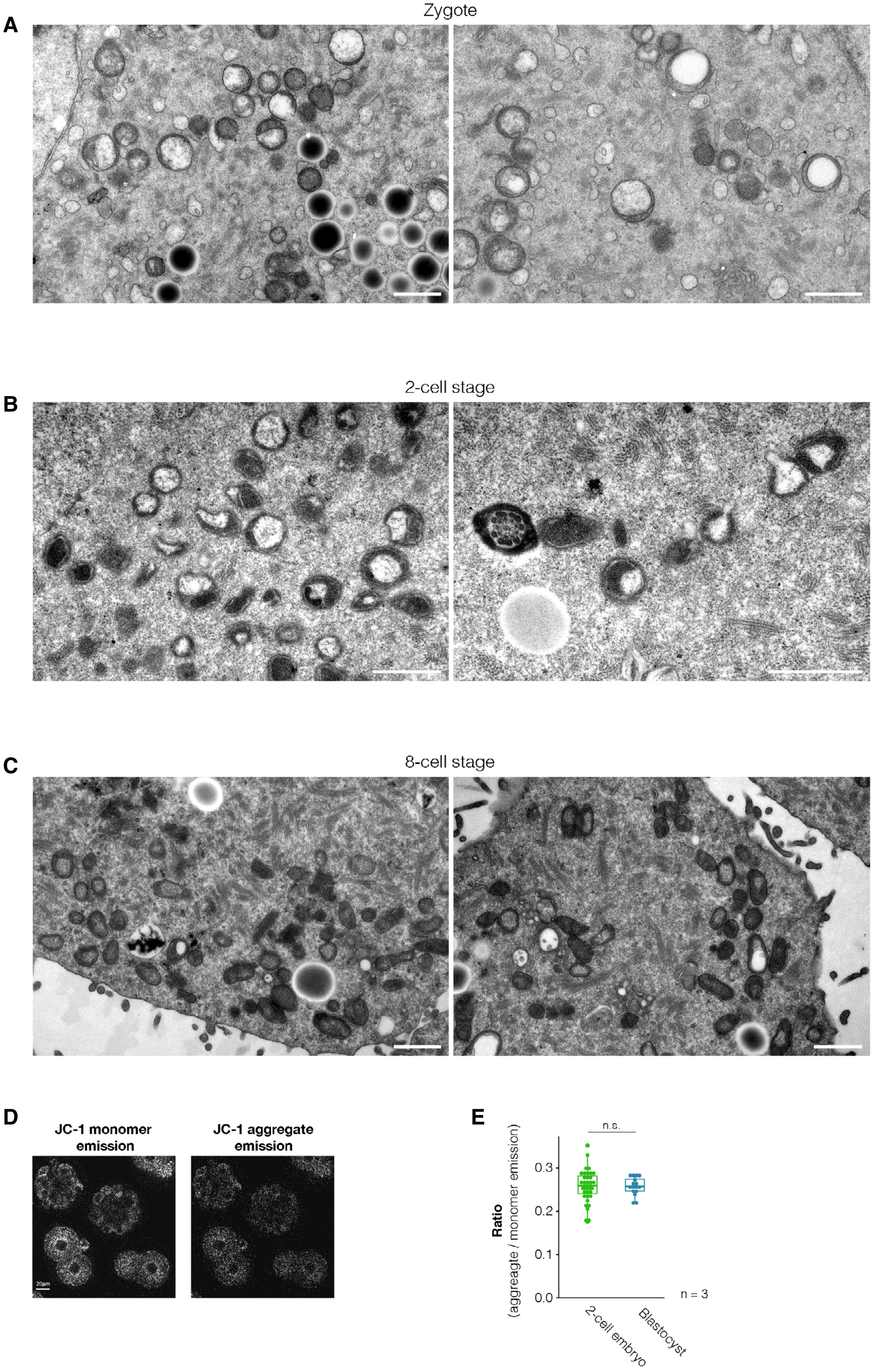

Figure EV4.

 © 2019 The Authors

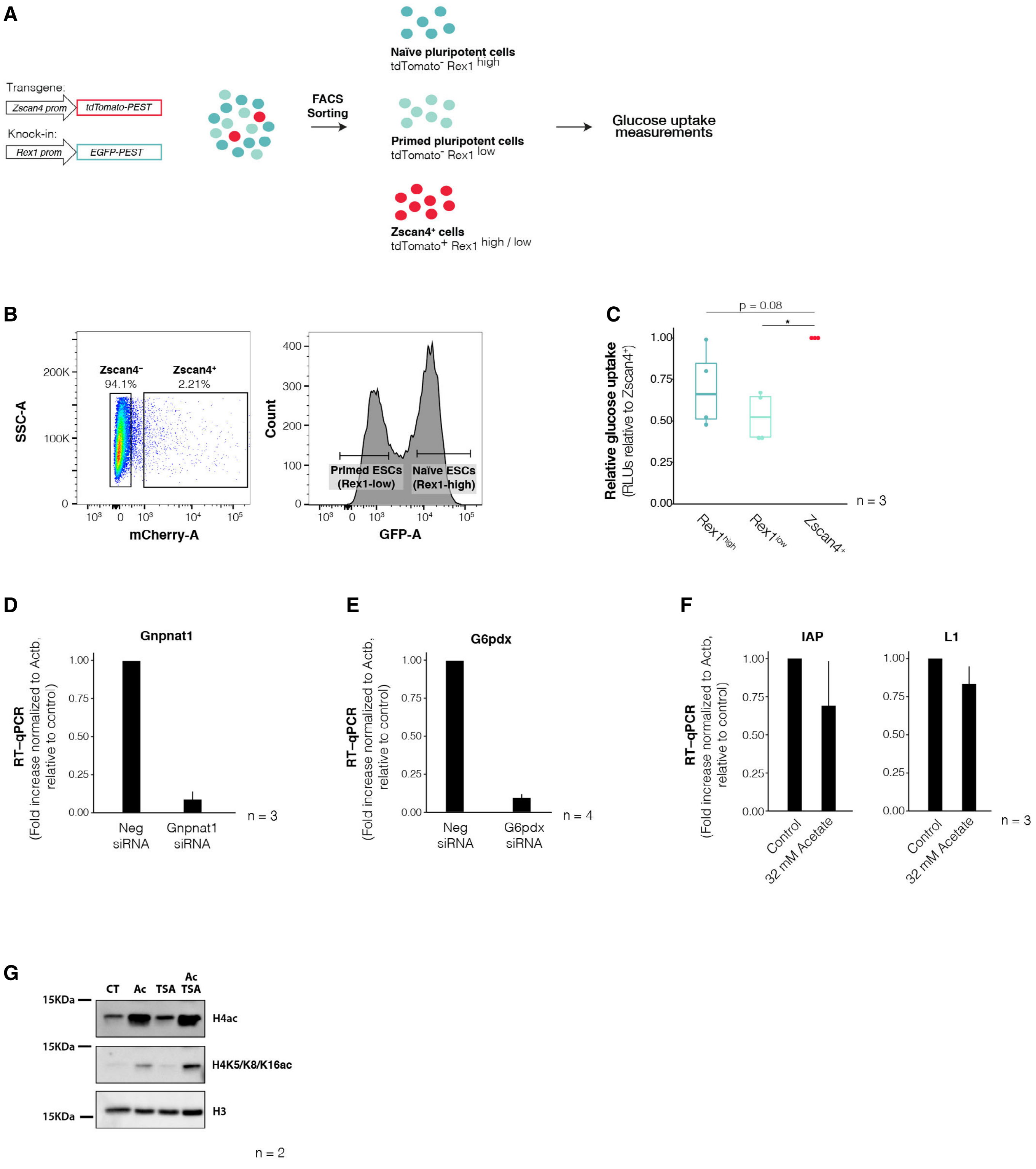
A
Transgene:
Zscan4 prom
tdTomato-PEST
Knock-in:
Rex1 prom
EGFP-PEST
FACS Sorting
Naïve pluripotent cells
tdTomato⁻ Rex1 high
Primed pluripotent cells
tdTomato⁻ Rex1 low
Zscan4⁺ cells
tdTomato⁺ Rex1 high / low
Glucose uptake measurements
B
SSC-A
Zscan4⁻
94.1%
Zscan4⁺
2.21%
mCherry-A
Count
Primed ESCs (Rex1-low)
Naïve ESCs (Rex1-high)
GFP-A
C
Relative glucose uptake (RLUs relative to Zscan4⁺)
p = 0.08
*
n = 3
Rex1high
Rex1low
Zscan4+
D
Gnpnat1
RT-qPCR (Fold increase normalized to Actb, relative to control)
Neg siRNA
Gnpnat1 siRNA
n = 3
E
G6pdx
Neg siRNA
G6pdx siRNA
n = 4
F
IAP
L1
Control
32 mM Acetate
n = 3
G
CT
Ac
TSA
Ac TSA
15KDa
H4ac
H4K5/K8/K16ac
H3
n = 2

Figure EV5.

Figure EV5. Zscan4$^+$ cells exhibit higher glucose uptake than both naïve and primed ES cells.

A Experimental design. ES cells were cultured in serum/LIF conditions over feeders for at least 5 days in the absence of 2i, and subsequently FACS-sorted into naïve pluripotent, primed pluripotent or Zscan4$^+$ cells. Glucose uptake rates were measured thereafter. Reporter constructs employed to identify all three distinct populations are represented on the left. An EGFP reporter driven by the Rex1 endogenous promoter was used to distinguish between Rex1-high (naïve pluripotent) and Rex1-low (primed pluripotent) cells, and a tdTomato cassette expressed downstream of an ectopic Zscan4c promoter was used to mark Zscan4$^+$ cells.

B Representative sorting gates used for the isolation of naïve pluripotent, primed pluripotent or Zscan4$^+$ cells. Zscan4$^+$ cells were defined as those positive for Zscan4c::tdTomato reporter (left), irrespective of their Rex1-EGFP fluorescence level. Naïve pluripotent and primed pluripotent stem cells were gated based on the bimodality of the Rex1-EGFP distribution (right).

C Glucose uptake rates in Zscan4$^+$ cells (red), naïve pluripotent stem cells (dark blue) and primed pluripotent stem cells (light blue) were measured using a luciferase-based assay across three independent biological replicates. Measurements are represented relative to the levels of Zscan4$^+$ cells. Boxes indicate the range between the first and third quartile, the band specifies the median, and the whiskers span the range of the data while extending no further than 1.5 times the interquartile range. Individual dots indicate the measurements obtained in each technical replicate. *$P < 0.05$; one sample *t*-test.

D, E RT–qPCR analysis of the indicated genes after transfection with the corresponding siRNAs. Shown are mean values ± s.d. of two technical replicates from three independent biological replicates.

F RT–qPCR of the indicated repeats upon 24 h of sodium acetate treatment. Shown are the mean ± s.d. of three independent cell cultures, performed in two technical replicates.

G Western blot for the indicated antibodies in lysates derived from control and acetate- and/or TSA-treated cultures.

Source data are available online for this figure.

 © 2019 The Authors
