# Supplementary figures and images for: A distinct metabolic state arises during the emergence of 2‐cell‐like cells
Source: EMBO Rep. 2019 Dec 18;21(1):e48354. doi: 10.15252/embr.201948354 (PMC6944916; doi:10.15252/embr.201948354)

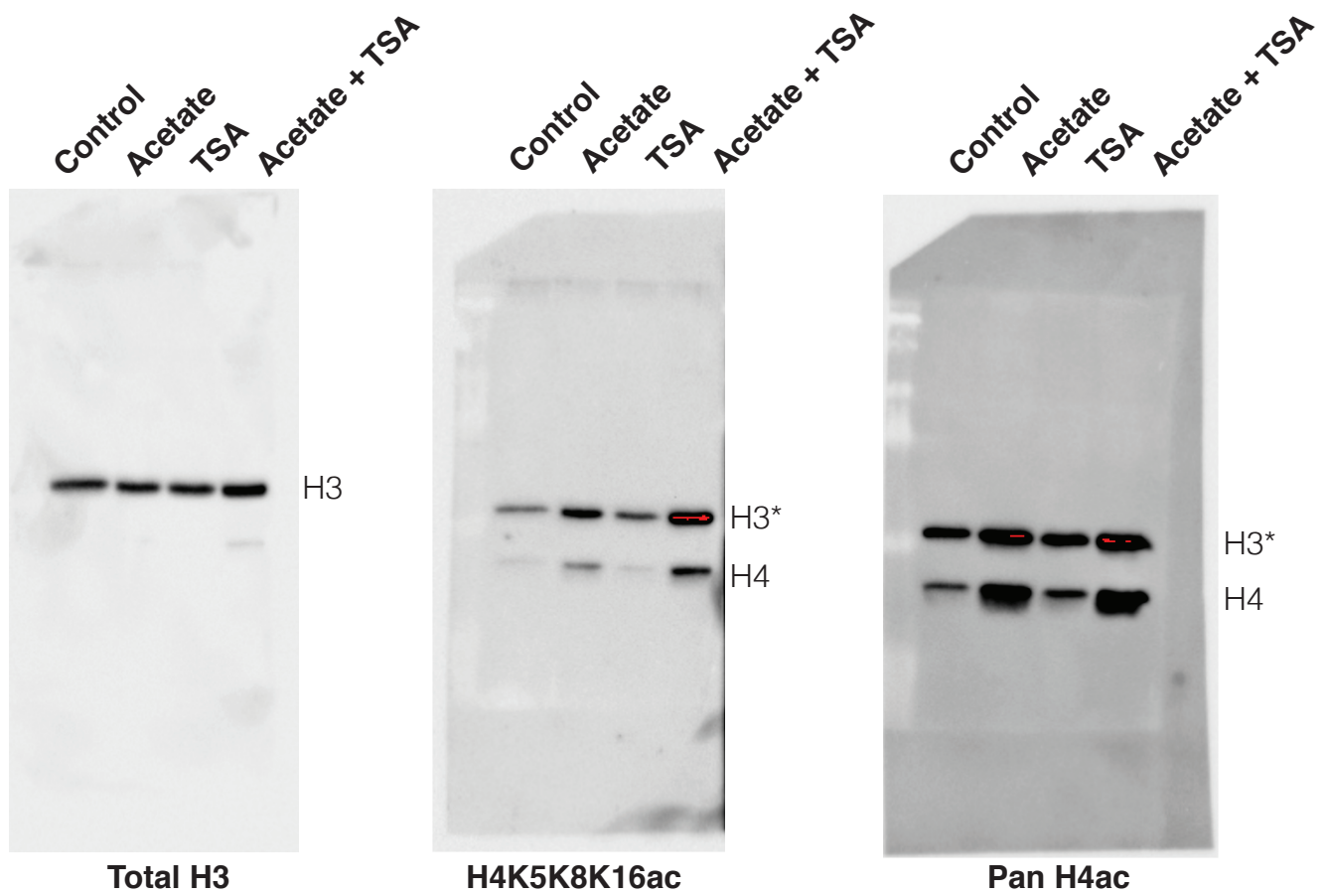

\*Non-specific band – corresponding to H3 size – recognised by the antibodies.

Supplement: Supplementary file 3 — Source Data for Expanded View [file EMBR-21-e48354-s007.zip › Source_Data_for_EV_Figures/Source_Data_for_FigEV5/Source_Data_for_FigEV5G.pdf]
