# Supplementary material for: A distinct metabolic state arises during the emergence of 2‐cell‐like cells
Source: EMBO Rep. 2019 Dec 18;21(1):e48354. doi: 10.15252/embr.201948354 (PMC6944916; doi:10.15252/embr.201948354)
Supplement: Supplementary file 4 — Review Process File [file EMBR-21-e48354-s008.pdf]

EMBO
reports

A distinct metabolic state arises during the emergence of 2-cell-like cells

Diego Rodriguez-Terrones, Götz Hartleben, Xavier Gaume, André Eid, Manuel Guthmann, Ane Iturbide and Maria-Elena Torres-Padilla

Review timeline:

Submission date:	26 April 2019
Editorial Decision:	19 June 2019
Revision received:	4 October 2019
Editorial Decision:	11 November 2019
Revision received:	13 November 2019
Accepted:	15 November 2019

Editor: Esther Schnapp

Transaction Report:

No Peer Review Process File is available with this article, as the authors have chosen not to make the review process public.
